# Lentinoids A–D, New Natural Products Isolated from *Lentinus strigellus*

**DOI:** 10.3390/molecules23040773

**Published:** 2018-03-28

**Authors:** Roger Vásquez, Nivia Rios, Godofredo Solano, Luis Cubilla-Rios

**Affiliations:** 1 Laboratory of Tropical Bioorganic Chemistry, Faculty of Natural, Exact Sciences and Technology, University of Panama, Panama 0824, Panama; royi071123@gmail.com; 2 Department of Microbiology, Faculty of Natural, Exact Sciences and Technology, University of Panama, Panama 0824, Panama; toxogondii@gmail.com; 3 Centro de Investigaciones en Productos Naturales (CIPRONA), Universidad de Costa Rica, San José 2060, Costa Rica; godofredo.solano@gmail.com; 4 Smithsonian Tropical Research Institute, Unit 0948, APO AA 34002–0948, Panama 0843, Panama

**Keywords:** *Lentinus strigellus*, lentinoids, antibacterial, panepoxydone, isopanepoxydone

## Abstract

Four novel lentinoids (**1**–**4**), along with the known compounds striguellone A (**5**), isopanepoxydone (**6**) and panepoxydone (**7**), were isolated as part of our studies on *Lentinus strigellus*. The structures of **1**–**4** have been established by 1D- and 2D-NMR and MS analysis. Compounds (**1**–**3**) and (**5**–**7**) were tested against *Listeria monocytogenes*, *Enterococcus faecalis*, *Pseudomonas aeruginosa* and *Klebsiella pneumoniae*. These compounds showed inhibition diameters ranging from 7.5–9.5 mm, however, when the minimum inhibitory concentration (MIC) was determined, only compound **1** showed a significant activity of 200 μg/mL. Intermediates for the biosynthesis of the oxygenated cyclohexenyl derivatives isolated from lentinoid fungi (genera *Lentinus* and *Panus*) are proposed.

## 1. Introduction

Fungi represent one of the most extensive groups of organisms and it is estimated that there are between 1.5 and 5.1 million species [1]. Their capability to produce secondary metabolites with a wide spectrum of biological activities causes a high level of interest in the scientific community [2]. Some of them are edible and/or have been used in traditional medicine, such as the fungus *Lentinus strigellus* [3]. In their initial stage of growth, they can sometimes be manipulated to generate new compounds, and they could have interesting properties for biological applications.

The genera *Lentinus* and *Panus* have some similarities that have generated controversy in some cases [4]. *L. strigellus* has also been described as *Panus strigellus* and *Panus rudis* var. *strigellus* [5]. Compounds such as strigellone A (**5**) [3], isopanepoxydone (**6**) [6], panepoxydone (**7**) [6], lentinoid E (**8**) [7] and other analogues have been reported from *L. strigellus* [8]. The lentinoids reported here have structural similarities with compounds isolated from species of the genera *Panus* (*rudis* and *conchatus* [6]) and *Lentinus* (*strigosus* [9] and *connatus* [10]). The fundamental structural scaffold of these compounds consists of an isoprenyl residue connected to a polyoxygenated cyclohexenyl unit. Lentinoid E (**8**) was previously isolated from the dichloromethane fraction obtained from a culture of *L. strigellus* grown in Sabouraud Dextrose Agar (SDA) adjusted to pH = 4.6, and using CaCl_2_ as elicitor at 26 °C for 15 days [7].

**Figure 1:**
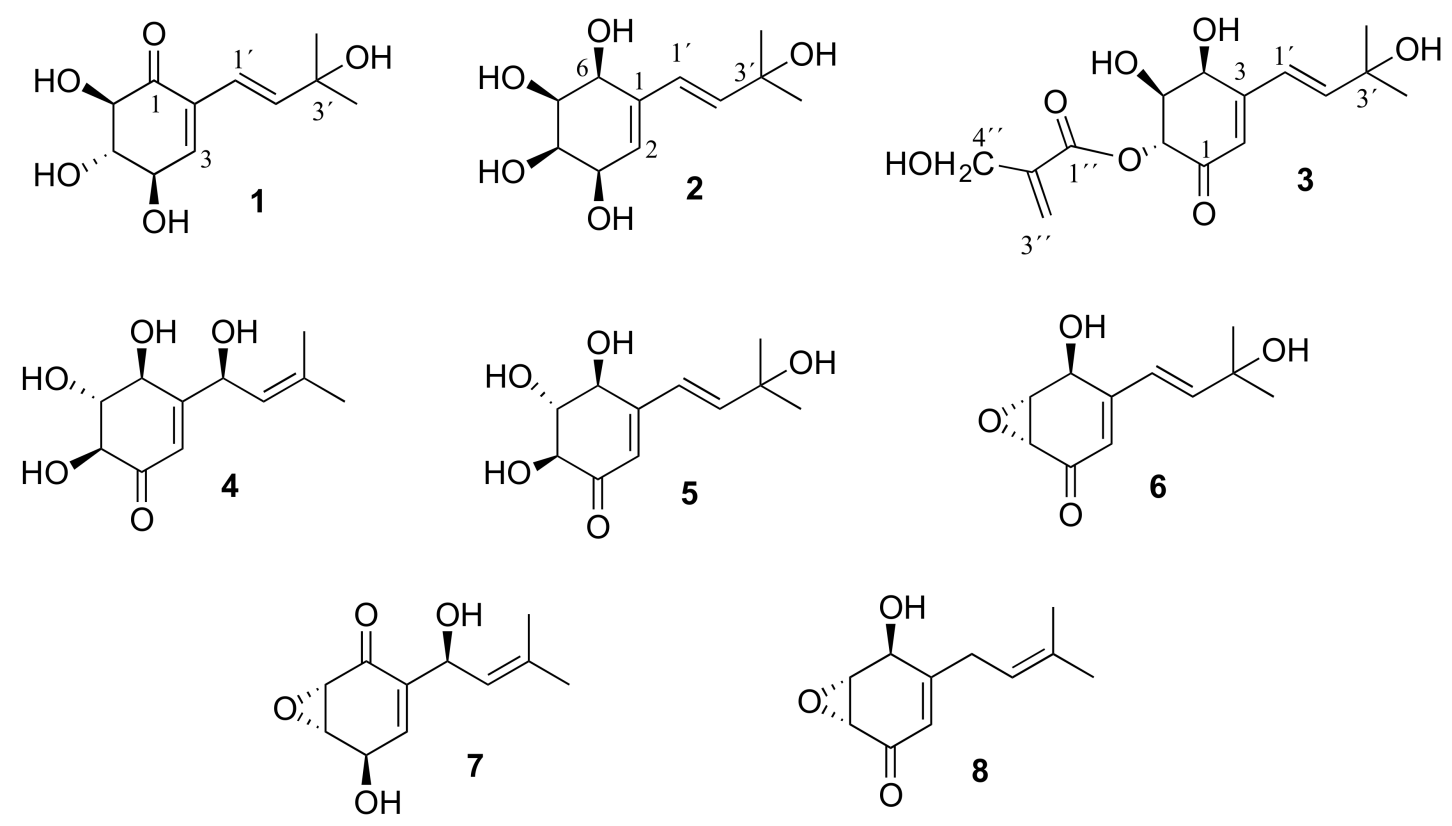
Structures of compounds **1**–**8**.

Continuing our study of *L. strigellus*, we undertook the analysis of the ethyl acetate fraction of the original extract using flash column chromatography and high performance liquid chromatography (HPLC). Thus, we describe the isolation and structural determination of four new lentinoids (**1**–**4**) along with known compounds (**5**–**7**) (Figure 1) that showed antibacterial properties.

## 2. Results and Discussion

Compound **1** was obtained as a brown viscous liquid. Its molecular formula, C_11_H_16_O_5_, was determined by High-resolution electrospray ionisation time-of-flight mass spectrometry (HR-ESI-TOF-MS) analysis on the basis of its cationized molecular peak [M + Na]^+^ at *m*/*z* 251.0891. The ion at *m*/*z* 269.0568 (100%) corresponded to [M + H_2_O + Na^+^] and the tandem mass spectrometry (MS^2^) spectrum showed that this ion is derived from the cluster [2M + H_2_O + Na^+^], *m*/*z* 497.0824. The Infrared (IR) spectrum showed bands at 3350 and 1690 cm^−1^ corresponding to a hydroxyl and carbonyl group, respectively. The nuclear magnetic resonance (NMR) data (Table 1) revealed the existence of an isoprenyl unit: two methyl groups (δ_H_ 1.30 (s); δ_C_ 29.7), a quaternary sp^3^ oxycarbon (δ_C_ 71.4), and two methines sp^2^ (δ_H_ 6.36, 6.44 (d); δ_C_ 120.1, 143.1). These signals have been observed in striguellone A (**5**) [3], a compound previously isolated from a culture of *L*. *strigellus*. This was confirmed by the correlation spectroscopy (COSY) experiment.

In the ^13^C-NMR spectrum, there was a quaternary carbon at δ_C_ 191.8 indicative of an α,β-unsaturated ketone. The chemical shift for the olefinic proton at C-3 displayed low-field shift at 6.89 ppm and appeared as a doublet, due to the coupling between H-3 and H-4, while in striguellone A, this appears as a singlet at 6.04 ppm (C-2) due the absence of this coupling.

**Figure 2:**
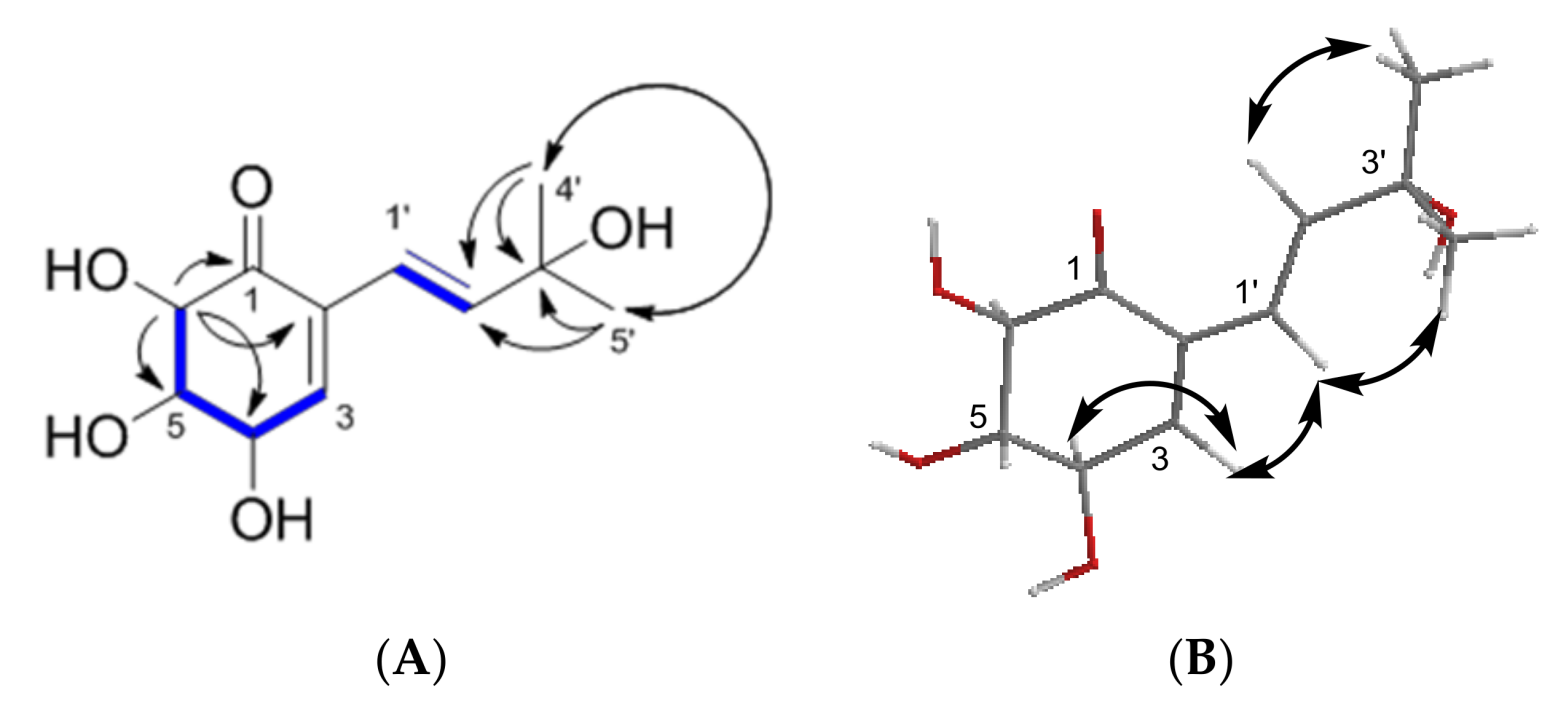
(**A**) Correlation spectroscopy (COSY) (in blue boldface), heteronuclear multiple-bond correlation (HMBC) (black arrows) and (**B**) Overhauser-effect spectroscopy (NOESY) (black arrows) of **1**.

The quaternary olefinic carbon (δ_C_ 135.2) at the α-position of the α,β-unsaturated ketone displayed a high-field shift in comparison to C-3 (δ_C_ 155.7) in striguellone A; additionally, the methine carbon (δ_C_ 145.4) appeared at low-field shift occupying the β-position. The COSY spectrum revealed correlations between H-3/H-4, H-4/H-5, H-5/H-6 and H-1′/H-2′ (Figure 2A). The position of the carbonyl group was confirmed by the heteronuclear multiple-bond correlation (HMBC) experiment (Figure 2A). The nuclear Overhauser-effect spectroscopy (NOESY) experiment showed, clearly, correlation of H-1′/H-2′ with H-3 and H-4′/H-5′. On the other hand, a correlation between H-3 and H-4 was observed (Figure 2B). No interaction was detected for H-4/H-5 and H-5/H-6, placing them, in both cases, on opposite sides, which was confirmed by the coupling constants (3.6 and 10.8 Hz, respectively). Thereafter, we have assigned the new name lentinoid A to compound **1**.

**Figure 3:**
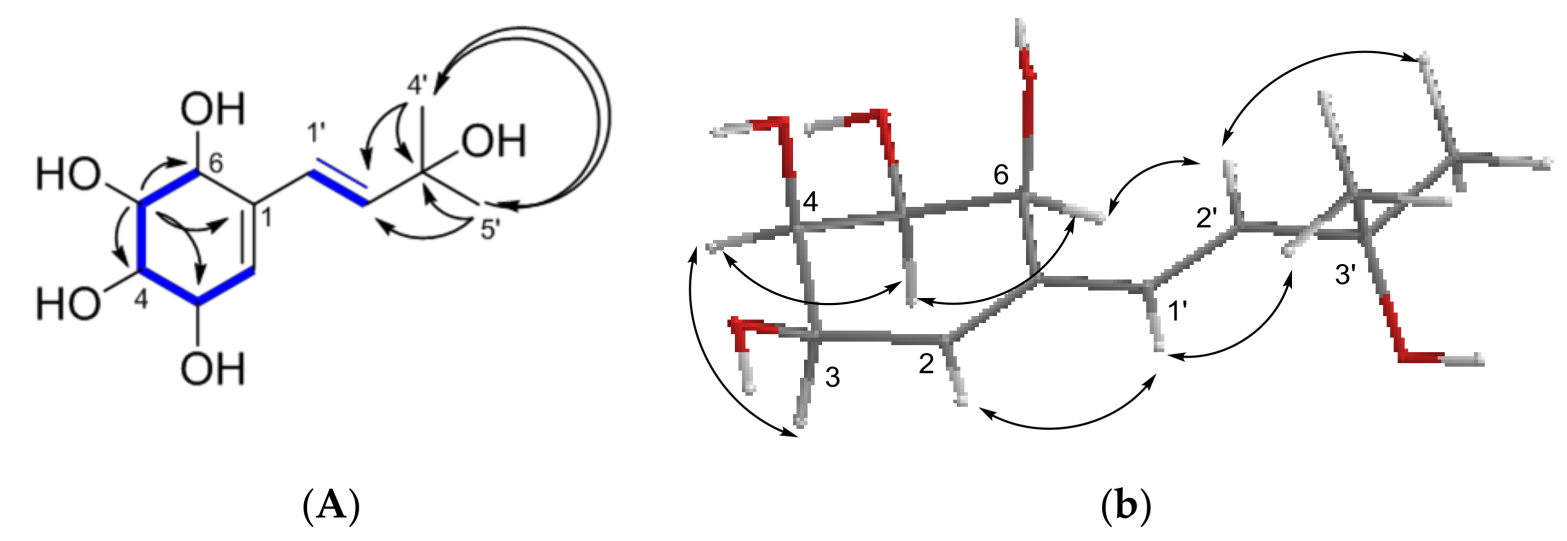
(**A**) COSY (in boldface), HMBC (black arrows) and (**B**) NOESY (black arrows) of **2.**

For compound **2**, a brown viscous liquid, the molecular formula C_11_H_18_O_5_ was assigned, which has three degrees of unsaturation according to its HR-ESI-TOF-MS data ([M + Na]^+^, *m/z* 253.1034, calcd. 253.1046 for C_11_H_18_O_5_Na). The ion at *m/z* 235.0940 (100%) in the mass spectrum corresponded to the loss of water [M + Na − H_2_O]^+^, which was confirmed by an MS^2^ experiment. The IR spectrum showed a band at 3300 cm^−1^ corresponding to hydroxyl groups, although no signal for a carbonyl group was observed. The COSY and HMBC experiments (Figure 3A) confirmed the presence of an isoprenyl unit as in compound **1** (δ_H_ 1.30 (s); δ_C_ 29.9), a quaternary sp^3^ oxycarbon (δ_C_ 71.4) and two sp^2^ methines (δ_H_ 6.15, 6.24 (d); δ_C_ 126.5, 140.9). Above, **2** showed a quaternary sp^2^ carbon (δ_C_ 136.5), an sp^2^ methine (δ_H_ 5.67 (br t); δ_C_ 125.9) and four sp^3^ oxymethines (δ_H_ 3.37 (t), 3.49 (br m), 4.35 (d), 4.59 (br d); δ_C_ 57.1, 56.0, 63.9, 65.3).

The union of the oxymethines was established based on the COSY experiment (Figure 3A), observing correlations between H-2/H-3, H-3/H-4, H-4/H-5 and H-5/H-6, and the corresponding coupling constants. The relative stereochemistry was determined taking into account the following NOESY correlations: H-3/H-4, H-4/H-5 and H-5/H-6 (Figure 3B and Figure S2.6), as well as the values of the coupling constants (all bellow 4.6 Hz) between the hydrogens of the cyclohexyl unit. Therefore, all the hydroxyl groups were determined to be on the same site of the molecule. See ^1^H- and ^13^C-NMR data in Table 1. We have assigned the new name lentinoid B to compound **2**.

For lentinoid C (**3**), the molecular formula C_15_H_20_O_7_ was assigned with six degrees of unsaturation according to its HR-ESI-TOF-MS data, [M + Na]^+^, *m/z* 335.1109 (calcd. 335.1101 for C_15_H_20_O_7_Na), with the bimolecular ion presented at *m/z* 647.2311. The IR spectrum showed a band at 3400 cm^−1^ corresponding to an OH group, and two bands for carbonyl groups: 1632 cm^−1^ for an α,β-unsaturated ketone and 1649 cm^−1^ for an α,β-unsaturated ester. Compound **3** showed similar NMR data (Table 2) to striguellone A, including the prenyl unit (two methyls (δ_C_ 29.6 and 29.7), an oxygenated quaternary carbon (δ_C_ 71.6), two sp^2^ carbons (δ_C_ 126.6 and 149.5)) and the α,β-unsaturated ketone (carbonyl group (δ_C_ 194.9), an sp^2^ quaternary carbon (δ_C_ 156.9) and sp^2^ methyne (δ_C_ 125.9)). Moreover, three oxygenated sp^3^ carbons were present (δ_C_ 76.6, 71.8 and 67.9). Altogether, the signals resemble the prenyl group and the cyclohexenyl ketone characteristic of lentinoids.

**Figure 4:**
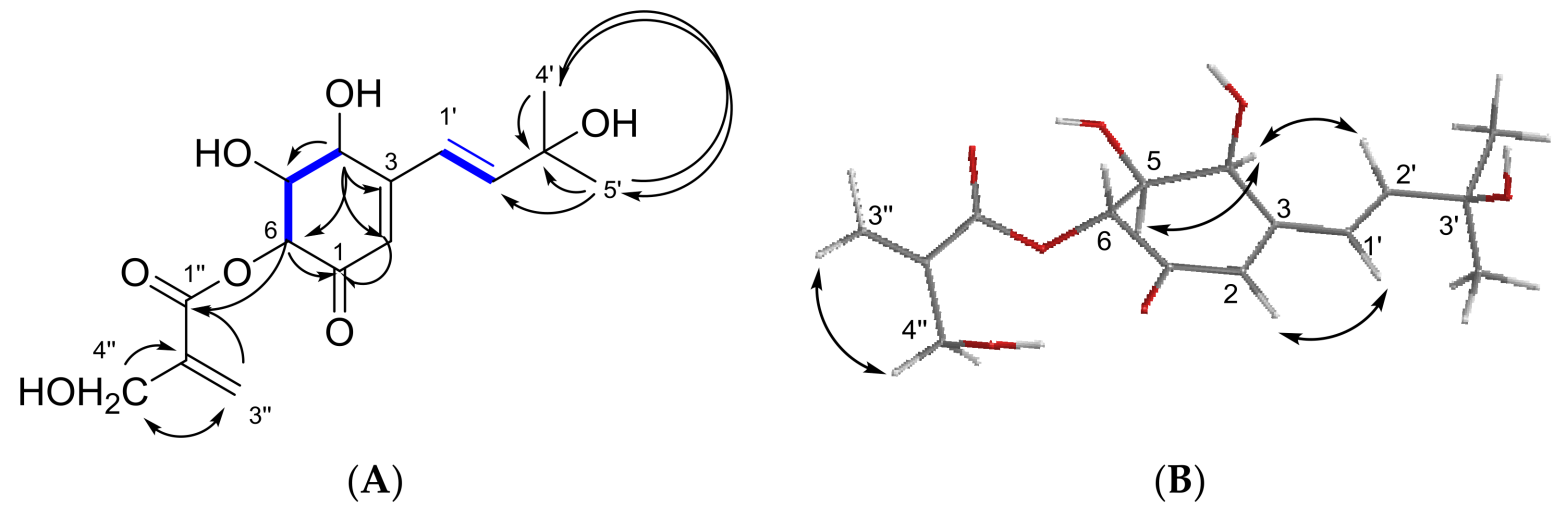
(**A**) COSY (in blue boldface), HMBC (black arrows) and (**B**) ROESY (black arrows) of **3**.

Additionally, the NMR data revealed the existence of a terminal methylene (δ_C_ 125.5, δ_H_ 5.96 and 6.35), a hydroxymethylene (δ_C_ 61.6, δ_H_ 4.34) and two quaternary carbons (δ_C_ 141.6 and δ_C_ 167.0), with the last one characteristic of a carboxyester group. The 2D NMR data (Figure 4A) showed the correlations between all protons and carbons for this fragment of compound **3**. Moreover, the connection of the carboxy group and the cyclohexenyl ring was established through the correlation of H-6 (δ_H_ 5.72, δ_C_ 76.6) to both carbonyl groups (δ_C_ 167.0 and 194.9) in equal intensity.

The rotating frame overhauser effect spectroscopy (ROESY) experiment (Figure 4B) showed correlation between H-4 and H-5, indicating that they are on the same site of the molecule, and this was confirmed by the coupling constant of 3.6 Hz. No correlation between H-5 and H-6 was observed, indicating that they are in axial positions with a coupling constant of 10.8 Hz. Thus, the relative stereochemistry of compound **3** was established.

Due to possible instability and the small amount of compound **4** obtained, the structure was characterized by its ^1^H-NMR spectrum. Compound **4** showed signals for the isoprenyl group as in panepoxydone (**7**) characterized by two methyl groups attached to an sp^2^ carbon (δ_H_ 1.69 and 1.70), a methyne (δ_H_ 5.29, d, *J* = 8.8 Hz) and a hydroxymethyne (δ_H_ 5.00, d, *J* = 8.8 Hz). Based on the chemical shift for the other three hydroxymethynes, the coupling constants (H-6: δ_H_ 3.96, d, 12.0; H-5: δ_H_ 3.63, dd, 8.4 and 12.0; H-4: δ_H_ 4.36, d, 8.4), and the olefin proton (δ_H_ 6.89) with a chemical shift characteristic of an α-proton in an α,β-unsaturated ketone, we proposed a structure for the newly discovered lentinoid D (**4**).

**Scheme 1:**
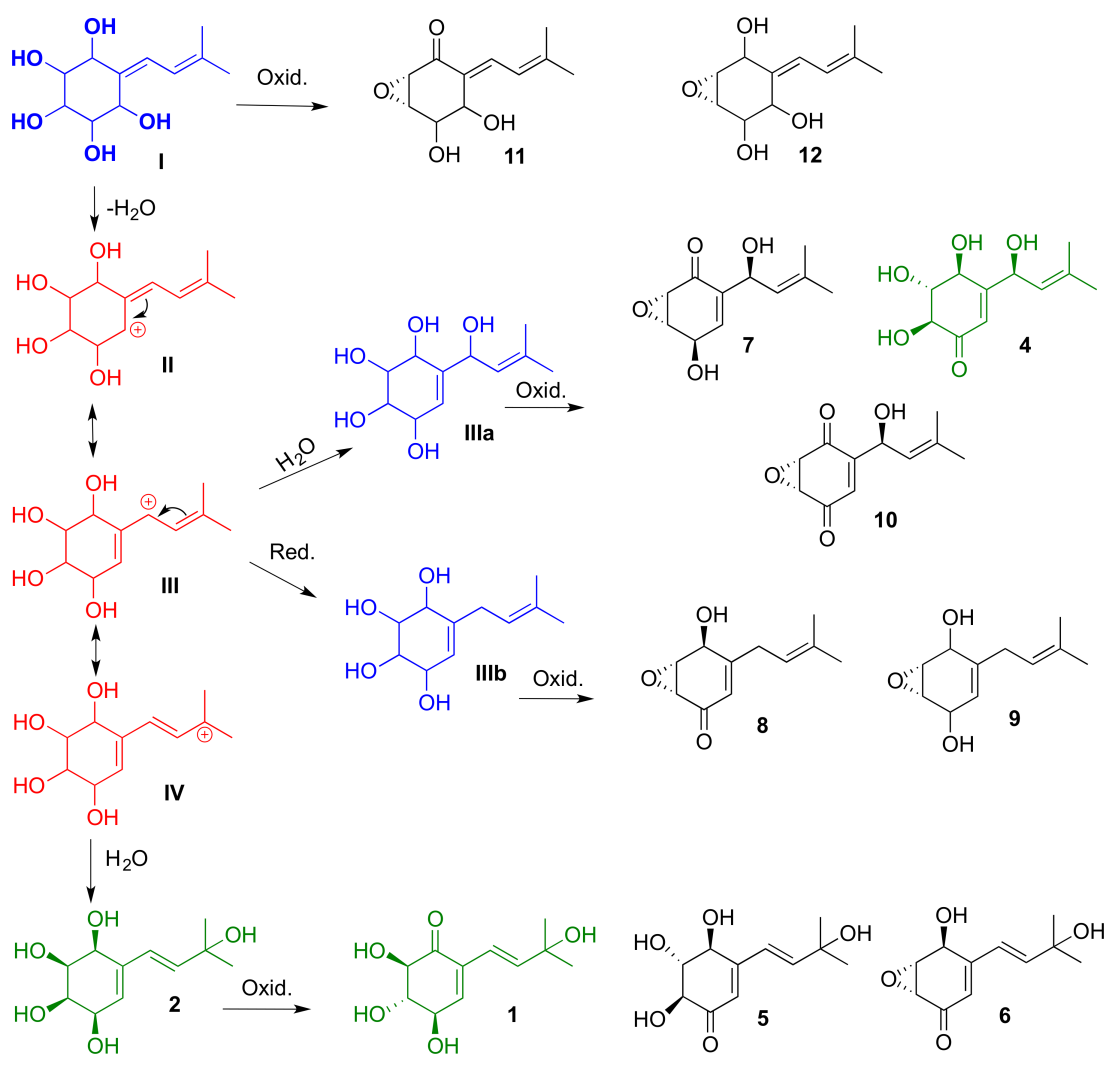
Proposed intermediates for the biosynthesis of the lentinoids, oxygenated cyclohexenyl derivatives, isolated from species of the genera *Lentinus* and *Panus*. Intermediates are in red. Compounds **1**–**2** and **4** are in green, the structure of not-yet-isolated compounds are in blue, and previously isolated compounds are in black: striguellone A (**5**), isopanepoxydone (**6**), panepoxydone (**7**), lentinoid E (**8**), 7-desoxypanepoxydol (**9**), panepoxydione (**10**), neopanepoxydone (**11**), and neopanepoxydol (**12**).

This group of compounds, isolated from lentinoid species (*Lentinus* and *Panus*), is characterized by an isoprenyl residue and an oxygenated cyclohexenyl ring, and could have similar biogenetic precursors. In Scheme 1, we proposed a not-yet-isolated precursor (**I**), and the structure of three possible intermediates (**II**–**IV**, in red) for the generation of this series of molecules. The biosynthesis may include two subsequent oxidation reactions of the not-yet-isolated compounds **I**, **IIIa**, **IIIb** and the newly reported lentinoid B (**2**). Compounds **I**, **IIIa** and **IIIb** (in blue) could be produced by some species of the genus *Lentinus* under different culture conditions.

### Antibacterial Activity

The bacterial growth inhibition was evaluated in vitro by the disc diffusion method [11]. Compounds **1**–**3** and **5**–**7** were tested at a concentration of 6.25 µg/µL against *Listeria monocytogenes, Enterococcus feacalis, Pseudomonas aeruginosa* and *Klebsiella pneumoniae.* The first three bacteria were inhibited by at least one of the compounds with an inhibition diameter (ID) ranging from 7.5–9.5 mm. No antibacterial activity was detected against *K. pneumoniae*. The positive control used was gentamycin sulfate (ID, 15–25 mm) at a concentration of 1.25 µg/µL (Table 3).

The minimum inhibitory concentration (MIC) was determined for compounds **1**, **2** and **3** using *E. faecalis* and *P. aeruginosa* (Table 4); nevertheless, only compound **1** resulted in a significant MIC; to inhibit *E. faecalis*, twice the amount of lentinoid A will be required. This could indicate that the presence of the carbonyl group is necessary for the antibacterial activity of lentinoid A compared to lentinoid B.

## 3. Materials and Methods

### 3.1. General

General experimental procedures. Optical rotations were measured in methanol on an Autopol V Plus Automatic Polarimeter (Whashington, NJ, USA) using a 10 cm cell. Ultra violet spectra were determined on a Waters 2996 PDA detector (Waters, Milford, MA, USA). IR spectra were determined using an Agilent Cary 630 FTIR spectrophotometer (film) (Agilent, Santa Clara, CA, USA). NMR spectra including 1D and 2D experiments were recorded at 400 MHz (JEOL, Ltd. Tokyo, Japan) and 600 MHz (Bruker, Billerica, MA, USA) using CD_3_OD and CDCl_3_ as solvents with tetramethylsilane (TMS) as internal standard. HRMS spectra were acquired on a Bruker micrOTOF-QIII (ESI-TOF), direct injection (Bruker, Billerica, MA, USA). The flash columns were made using silica gel 100 (70–230 mesh ASTM, Merck, Darmstadt, Germany) and silica gel (200–400 mesh, 60 Å, Sigma-Aldrich, St. Louis, MO, USA), the fractions collected were monitored by Sigma-Aldrich TLC plates 3110 (Sigma-Aldrich, St. Louis, MO, USA) and spots were detected using *p*-anisaldehyde spray reagent after heating. HPLC separations (Waters Delta 600) were carried out using a normal-phase semi-preparative column (YMC-Pack Sil, S-5 μm, 12 nm, 150 × 10 mm ID), a chiral column (QuiralPack IA, 5 μm 150 × 4.6 mm ID, Daicel Corporation, Tokyo, Japan) and a photodiode array detector Waters 2996. The injector was equipped with a 200 μL loop. Chemical structures (1D and 3D (force field MM2)) were drawn using Chemdraw (Perkin Elmer, Waltham, USA), version: 16.0.1.4.

### 3.2. Fungal Material

Fruiting bodies of *Lentinus strigellus* (collection number LC46) [7] were collected on burned wood near La Nevera highway in the Boquete, a region of Chiriquí Province in Panamá. The specimen was identified as *Lentinus strigellus* using molecular phylogenetic analyses. The air-dried specimens were deposited at the National Herbarium of the University of Panamá (PMA115711) and in the Herbarium of the Autonomous University of Chiriquí (UCH7481), Panamá.

### 3.3. Cultivation and Crude Extract

Initially, *L. strigellus* was cultivated in potato dextrose agar (PDA), malt extract agar (MEA) and Sabouraud Dextrose Agar (SDA) using six petri dishes each for 15 days. The chemical compositions of these three extracts were analyzed by thin-layer chromatography and NMR spectroscopy. A greater chemical diversity in the SDA extract using this analysis was detected. Hence, SDA cultivation was evaluated further, including the addition of the elicitors: arginine, glutamic acid, CaCl_2_, CuSO_4_ and FeSO_4_, and at three different pH values: 4.6, 5.6 and 6.6. The period of 15 days and temperature at 26 °C were the same for all experiments. The biomass yield (221 mg) was the highest for SDA with CaCl_2_ and a pH of 4.6 (calcium in low concentration tends to increase the yield of biomass produced [11]; in some cases this could bind proteins causing conformational changes and activating or inactivating mechanisms of catalysis [12]).

Culturing involving 67 petri dishes and the optimal conditions resulted in 3.10 g of crude ethyl acetate extract. To prepare the extract, the content of 15 petri dishes was transferred into one 1 L glass flask, with 400 mL of ethyl acetate, and extracted by sonicating for 20 min. This was repeated four times. The extract was recovered by drying under reduced pressure at 30 °C [7].

### 3.4. Compound Isolation

3.0 g of crude extract was fractionated by flash chromatography (4 cm ID) using silica gel (200–400 mesh, 10 g) and 1 L each of the following solvents: *n*-hexane, CH_2_Cl_2_, EtOAc, acetonitrile and water. All fractions (A–E) were analyzed by TLC. Fraction C (EtOAc), having a mass of 1561.4 mg (50.4%), was subjected to flash chromatography (2.5 cm ID) using silica gel (200–400 mesh, 70 g), and eluted with a gradient system from 100% CH_2_Cl_2_ to 100% EtOAc followed by a gradient from 100% EtOAc to 100% MeOH to afford 8 fractions (C1 to C8).

Fraction C6 (74.1 mg, 4.7%) was purified by normal-phase HPLC using a linear gradient (from 75:25 *n*-hexane–isopropanol to 40:60 *n*-hexane–isopropanol at 1.00 mL/min) in 55 min, to give 4 fractions, where the peak at *t*_R_ 17 min corresponded to panepoxydone (**7**) (8.1 mg, 10.9%) and the peak at *t*_R_ 21.5 min corresponded to isopanepoxydone (**6**) (12.3 mg, 16.6%).

Fraction C7 (235.0 mg, 15.1%) was flash chromatographed (2 cm ID) with silica gel (200–400 mesh, 22 g), and *n*-hexane/EtOAc (*v*/*v* = 100:0–0:100) and EtOAc/MeOH (*v*/*v* = 100:0–0:100) as eluents, to give 14 fractions (C7.1 to C7.14). C7.9 (76.2 mg, 32.4%) was subjected to normal-phase HPLC using a linear gradient (from 75:25 *n*-hexane–isopropanol to 40:60 *n*-hexane–isopropanol at 1.00 mL/min) in 55 min, to give 3 fractions, where the peak at *t*_R_ 18 min corresponds to striguellone A (**5**) and the peak at *t*_R_ 23 min corresponds to lentinoid A (**1**).

Fraction C7.10 (55.0 mg, 23.4%) was purified by normal-phase HPLC using a linear gradient (of 75:25 *n*-hexane–isopropanol to 40:60 *n*-hexane–isopropanol at 1.00 mL/min) in 55 min, to give 6 fractions (I–VI), where the peak at *t*_R_ 33 min (fraction VI) corresponds to lentinoid B (**2**) 7.4 mg (13.5%). Fraction V (26.0 mg, 47.3%) was purified by normal-phase HPLC using a linear gradient (85:15 to 30:70 *n*-hexane–isopropanol at 1.00 mL/min) in 55 min, to give 3 fractions (V.1 to V.3). V.1 (7.3 mg, 28.1%) was purified by HPLC (Chiral Pack IA, 5 µm, 150 × 4.6 mm ID) using the isocratic 85:15 *n*-hexane–isopropanol at 0.50 mL/min to give lentinoid C (**3**) (*t*_R_ 17 min; 2.6 mg, 35.6%). V.2 (5.4 mg, 20.8%) was purified by HPLC (Chiral Pack IA, 5 µm, 150 × 4.6 mm ID) using the isocratic 85:15 *n*-hexane–isopropanol at 0.50 mL/min to give lentinoid D (**4**) (*t*_R_ 12 min; 1 mg, 13.7%).

### 3.5. Lentinoid A *(**1**)*

Brown viscous liquid; ${\left[\alpha \right]}_{D}^{25}$ −64.5 (c 0.0029, MeOH); UV (MeOH) λ_max_ 273 and 212 nm; IR mmax: 3350, 2948, 2876, 1690, 1390 and 1040 cm^−1^; ^1^H- and ^13^C-NMR data, see Table 1; (+)-HR-ESI-TOF-MS *m*/*z* 251.0891 (calcd. 251.0890 for C_11_H_16_O_5_Na).

### 3.6. Lentinoid B *(**2**)*

Brown viscous liquid; ${\left[\alpha \right]}_{D}^{25}$ +3.3 (c 0.0024, MeOH); UV (Hx/IP) λ_max_ 233 nm; IR mmax: 3300, 2942, 2831 and 1021 cm^−1^; ^1^H- and ^13^C-NMR data, see Table 1; (+)-HR-ESI-TOF-MS *m*/*z* 253.1034 [M + Na]^+^ (calcd. 253.1046 for C_11_H_18_O_5_Na).

### 3.7. Lentinoid C *(**3**)*

Brown viscous liquid; ${\left[\alpha \right]}_{D}^{25}$ −75.0 (c 0.0005, MeOH); UV (MeOH) λ_max_ 278 and 208 nm; IR mmax: 3280, 2951, 2840, 1649, 1632 and 1013 cm^−1^; ^1^H- and ^13^C-NMR data, see Table 2; (+)-HR-ESI-TOF-MS *m*/*z* 335.1109 (calcd. 335.1101 for C_15_H_20_O_7_Na).

### 3.8. Antibacterial Activities [13,14]

The antibacterial activity was determined through the susceptibility test of the British Society for Antimicrobial Chemotherapy (BSAC).

#### 3.8.1. Preparation of 0.5 McFarland Turbidity Standard

The turbidity standard was prepared using 0.5 mL of solution of BaCl_2_ (BaCl_2_.2H_2_O; 0.048 M) in a tube containing 99.5 mL of H_2_SO_4_ (0.18 M). The absorbance of the McFarland standard (between 0.08 and 0.10 at 625 nm) was verified using a spectrophotometer. The solution was stored in the dark at 24 ± 2 °C.

#### 3.8.2. Preparation of Bacterial Inoculum

Each bacterium was cultivated in Tripticase Soy Agar (TSA) for 24 h; thereafter, five colonies were chosen and transferred into a tube containing saline and isotonic solution and then visually compared with the 0.5 McFarland standard.

#### 3.8.3. Bacterial Growth Inhibition in Vitro

The bacterial growth inhibition was evaluated in vitro by the disc diffusion method using Trypticase Soy Agar (TSA) in Petri dishes (145 mm) and placing a disc (6 mm diameter) with 50 µg of each tested compound dissolved in DMSO. The plate was then incubated for 18 h at 37 °C, and the inhibition area were measured. For the positive control, gentamycin sulfate (10 μg/mL) was used and the negative control was DMSO. The diameter of the inhibition around each disk was measured, providing a measure of zone–of-inhibition (ZOI) in mm (including the diameter of the disc).

#### 3.8.4. Minimum Inhibitory Concentration (MIC)

The minimum inhibitory concentration (MIC) was determined using a stock solution prepared by adding 300 µg of each compound in 3 mL of Trypticase Soy Broth (TSB). Serial dilutions of the evaluated compounds and control were performed to determine the MIC. The evaluated concentrations of each compound were 100.0, 40.0, 33.3, 11.1 and 5.6 μg/mL. Each solution was thereafter inoculated with 50 μL (0.5 McFarland) of a culture of *E. faecalis* and *P. aeruginosa*, and incubated at 37 °C for 18 h. The positive control (gentamycin sulfate 10 mg/mL) concentrations were similar to those of the compounds. Additionally, two blanks were used, one with culture medium alone and one with DMSO. Each assay was performed in duplicate.

## 4. Conclusions

In summary, we have characterized four new lentinoids produced by *L. strigellus* as a response to changes in culture conditions. They represent new additions to the chemical diversity produced by this fungus. The compounds represented in Scheme 1, isolated from the genera *Lentinus* and *Panus*, could have similar precursors. Additional improvements to the experimental culture conditions and the use of additional analytical tools could lead to the isolation of new compounds of this class and therefore new active molecules. Moreover, three of these compounds showed antibacterial activity in a bacterial growth inhibition assay (inhibition zone in mm). However, when the minimum inhibitory concentration (MIC) was determined, only compound **1** showed a significant activity of 200 μg/mL.

## Figures and Tables

**Table 1:** NMR data for compounds **1**–**2** in MeOH*-d*_4_, (*J* in Hz; ^1^H-NMR at 400 MHz; ^13^C-NMR at 100 MHz).

Pos.	1		2	
	δ_C_	δ_H_	δ_C_	δ_H_
1	191.8, C		136.5, C	
2	135.2, C		125.9, CH	5.67 (br t; 4.0)
3	145.4, CH	6.89 (d; 2.4)	63.9, CH	4.35 (d; 4.4)
4	73.1, CH	4.41 (dd; 2.4, 8.4)	57.1, CH	3.37 (t; 4.0)
5	79.3, CH	3.63 (dd; 8.4, 11.6)	56.0, CH	3.49 (br m)
6	68.8, CH	4.60 (d; 11.6)	65.3, CH	4.59 (br d; 4.6)
1′	120.1, CH	6.36 (d; 16.0)	126.5, CH	6.15 (d; 16.0)
2′	143.1, CH	6.44 (d; 16.0)	140.9, CH	6.24 (d; 16.0)
3′	71.4, C		71.4, C	
4′	29.7, CH_3_	1.30 (s)	29.9, CH_3_	1.30 (s)
5′	29.7, CH_3_	1.30 (s)	29.9, CH_3_	1.30 (s)

**Table 2:** NMR data for compound **3** in MeOH*-d*_4_, (*J* in Hz; ^1^H-NMR at 600 MHz; ^13^C-NMR at 150 MHz).

Pos.	δ_C_	δ_H_	Pos.	δ_C_	δ_H_
1	194.9		3´	71.6	
2	125.9	6.02 (1H, s)	4´	29.7	1.37 (3H, s)
3	156.9		5´	29.6	1.36 (3H, s)
4	67.9	4.72 (1H, d; 3.6)	1´´	167.0	
5	71.8	4.05 (1H, dd; 3.6, 10.8)	2´´	141.6	
6	76.6	5.72 (1H, d; 10.8)	3´´	125.5	5.96 (1H, m) 6.35 (1H, m)
1′	126.6	6.46 (1H, d; 16,0)	4´´	61.6	4.34 (2H, m)
2′	149.5	6.64 (1H, d; 16,0)			

**Table 3:** Antibacterial activity of compounds **1**–**3** and **5**–**7**, inhibition diameter (mm).

Bacteria Species	Compounds						Positive Control
	1	2	3	5	6	7	Gentamycin Sulfate
*Listeria monocytogenes*	I	I	I	I	7.5	I	25
*Enterococcus faecalis*	9	9	8	8	9.5	I	15
*Pseudomonas aeruginosa*	I	9.5	I	7.5	8.5	I	20

I = inactive.

**Table 4:** Antibacterial activities of compounds **1**–**3**, MIC (μg/mL).

Bacteria Species	Compounds			Positive Control
	1	2	3	Gentamycin Sulfate
*E. faecalis*	200	-----	-----	100
*P. aeruginosa*	-----	160	-----	33

---- No activity detected.

## Supplementary Materials

The following are available online, ^1^H- and ^13^C-NMR, 2D NMR and MS data for compounds **1**–**3** and ^1^H-NMR for compound **4**. The isolations chromatograms for each novel compound are present.
