# Supplementary material for: Lentinoids A–D, New Natural Products Isolated from Lentinus strigellus
Source: Molecules. 2018 Mar 28;23(4):773. doi: 10.3390/molecules23040773 (PMC6017980; doi:10.3390/molecules23040773)
Supplement: Supplementary file 1 [file molecules-23-00773-s001.pdf]

Supplementary data

Lentinoids A-D, new natural products isolated from *Lentinus strigellus*

Roger Vásquez[1], Nivia Rios[2], Godofredo Solano [3], Luis Cubilla-Rios[1,4]*

[1] Laboratory of Tropical Bioorganic Chemistry, Faculty of Natural and Exact Sciences and Technology, University of Panama, Panama; royi071123@gmail.com (R.V.); luis.cubilla@up.ac.pa (L.C.R.)

[2] Department of Microbiology, Faculty of Natural and Exact Sciences and Technology, University of Panama, Panama; toxogondii@gmail.com

[3] Centro de Investigaciones en Productos Naturales (CIPRONA), Universidad de Costa Rica. San José, Costa Rica; godofredo.solano@gmail.com

[4] Smithsonian Tropical Research Institute, Unit 0948, APO AA 34002-0948, Panama.

- Figure S1.1. ^{1}H NMR spectra (400 MHz, methanol-d_4) of compound **1**
- Figure S1.2. ^{13}C NMR spectra (100 MHz, methanol-d_4) of compound **1**
- Figure S1.3. ^{1}H- ^{13}C HSQC NMR data (in methanol-d_4) of compound **1**
- Figure S1.4. ^{1}H- ^{13}C HMBC NMR data (in methanol-d_4) of compound **1**
- Figure S1.5. ^{1}H-^{1}H COSY NMR data (in methanol-d_4) of compound **1**
- Figure S1.6. ^{1}H-^{1}H NOESY NMR data (in methanol-d_4) for compound **1**
- Figure S1.7. ESIMS (positive mode) data for compound **1**
- Figure S1.8. Chromatogram of compound **1**: **A)** Separation; **B)** Purity
- Figure S2.1. ^{1}H NMR spectra (400 MHz, methanol-d_4) of compound **2**
- Figure S2.2. ^{13}C NMR spectra (100 MHz, methanol-d_4) of compound **2**
- Figure S2.3. ^{1}H- ^{13}C HSQC NMR data (in methanol-d_4) of compound **2**
- Figure S2.4. ^{1}H- ^{13}C HMBC NMR data (in methanol-d_4) of compound **2**
- Figure S2.5. ^{1}H-^{1}H COSY NMR data (in methanol-d_4) of compound **2**
- Figure S2.6. ^{1}H-^{1}H NOESY NMR data (in methanol-d_4) for compound **2**
- Figure S2.7**.** Expansion of ^{1}H-^{1}H NOESY NMR for the correlations between H4-H5 in compound **2**
- Figure S2.8. ESIMS (positive mode) data for compound **2**
- Figure S2.9. Chromatogram of compound **2**: **A)** Separation; **B)** Purity
- Figure S3.1. ^{1}H NMR spectra (600 MHz, methanol-d_4) of compound **3**
- Figure S3.2. ^{13}C NMR spectra (150 MHz, methanol-d_4) of compound **3**
- Figure S3.3. ^{1}H- ^{13}C HSQC NMR data (in methanol-d_4) of compound **3**
- Figure S3.4. ^{1}H- ^{13}C HMBC NMR data (in methanol-d_4) of compound **3**
- Figure S3.5. ^{1}H-^{1}H COSY NMR data (in methanol-d_4) of compound **3**
- Figure S3.6. ^{1}H-^{1}H ROESY NMR data (in methanol-d_4) for compound **3**
- Figure S3.7. ESIMS (positive mode) data for compound **3**
- Figure S3.8**. A)** Normal phase HPLC separation chromatogram with semi-preparative column for compound **3. B)** Normal phase HPLC separation chromatogram with chiral pack column for compound **3**
- Figure S4.1. ^{1}H NMR spectra (400 MHz, methanol-d_4) of compound **4**
- Figure S4.2**. A)** Normal phase HPLC separation chromatogram with semi-preparative column for compound **4. B)** Normal phase HPLC separation chromatogram with chiral pack column for compound **4**

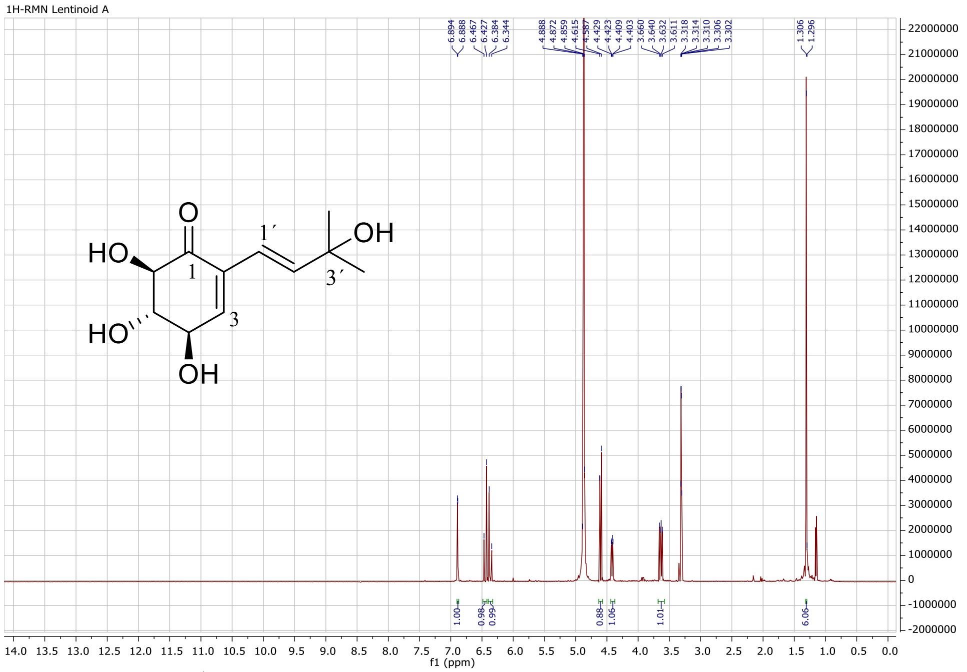

Figure S1.1. ^{1}H NMR spectra (400 MHz, methanol-d_4) of compound **1**

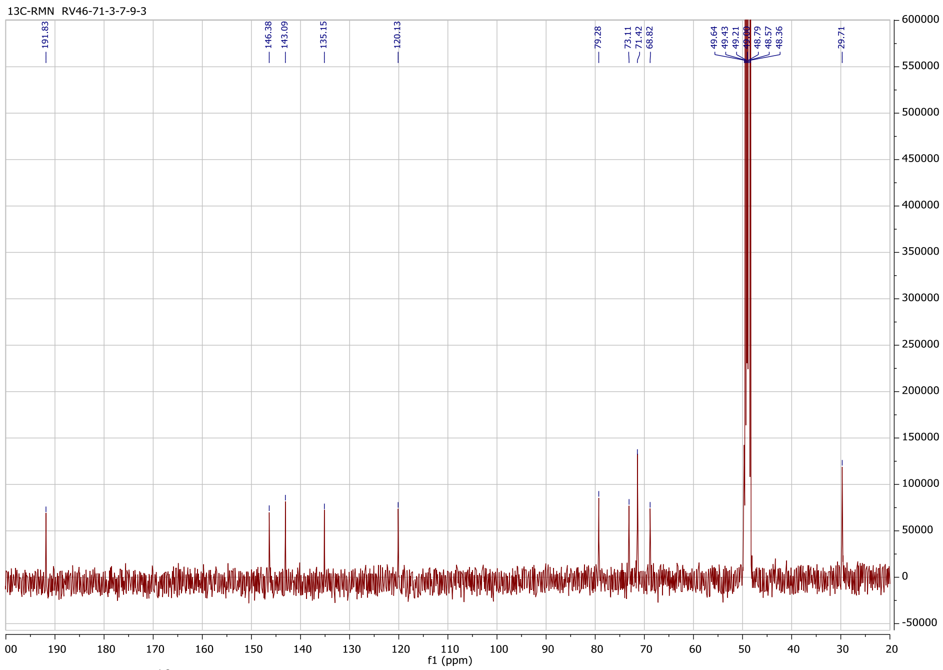

Figure S1.2. ^{13}C NMR spectra (100 MHz, methanol-d_4) of compound **1**

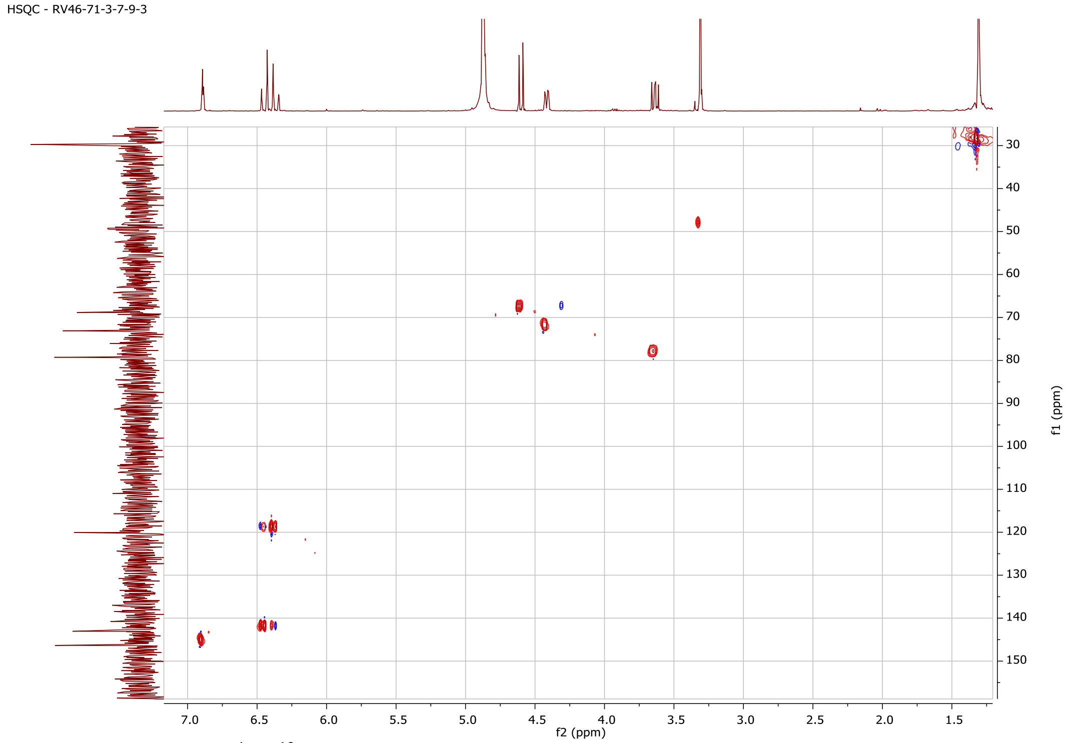

Figure S1.3. ^{1}H- ^{13}C HSQC NMR data (in methanol-d_4) of compound **1**

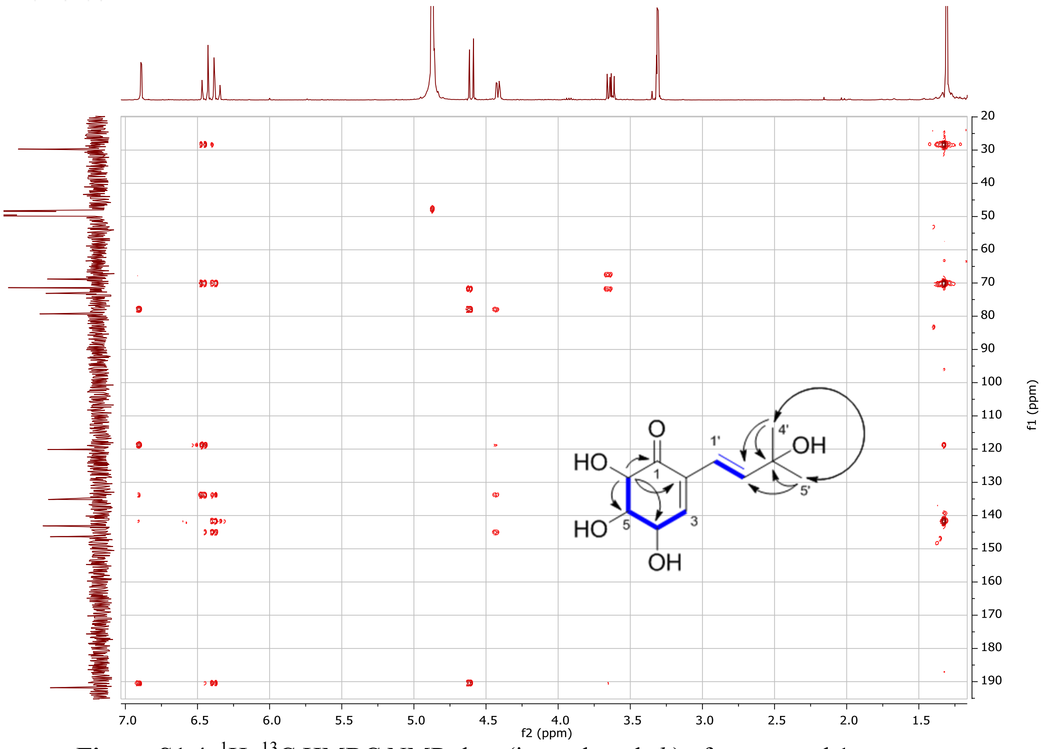

Figure S1.4. ^{1}H- ^{13}C HMBC NMR data (in methanol-d_4) of compound **1**

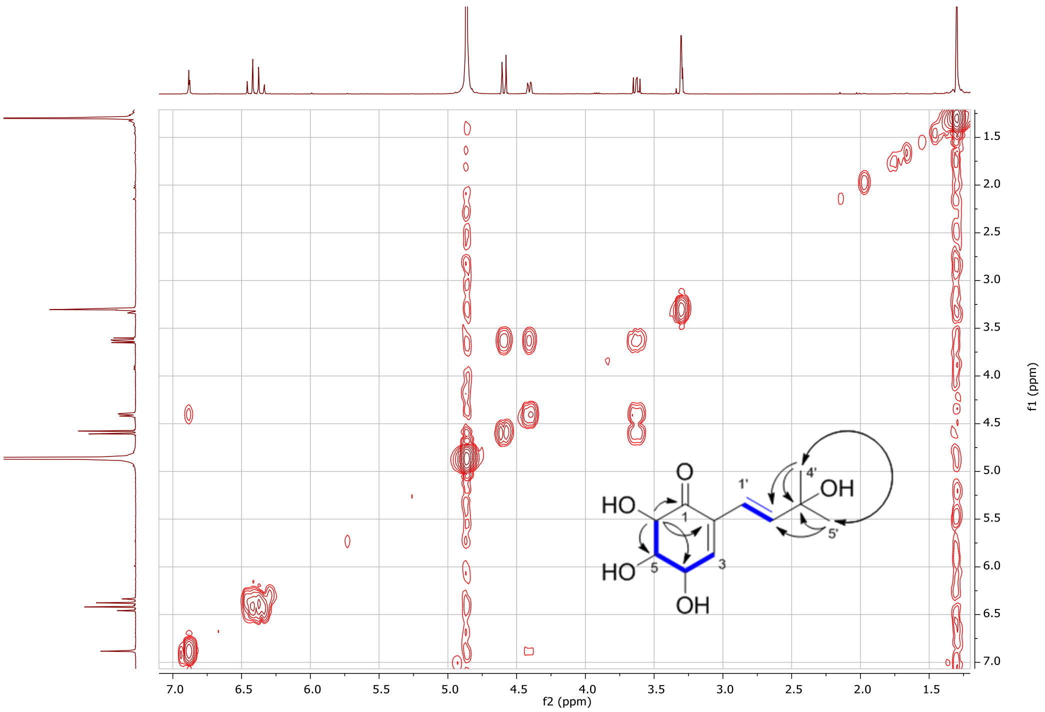

Figure S1.5. ^{1}H-^{1}H COSY NMR data (in methanol-d_4) of compound **1**

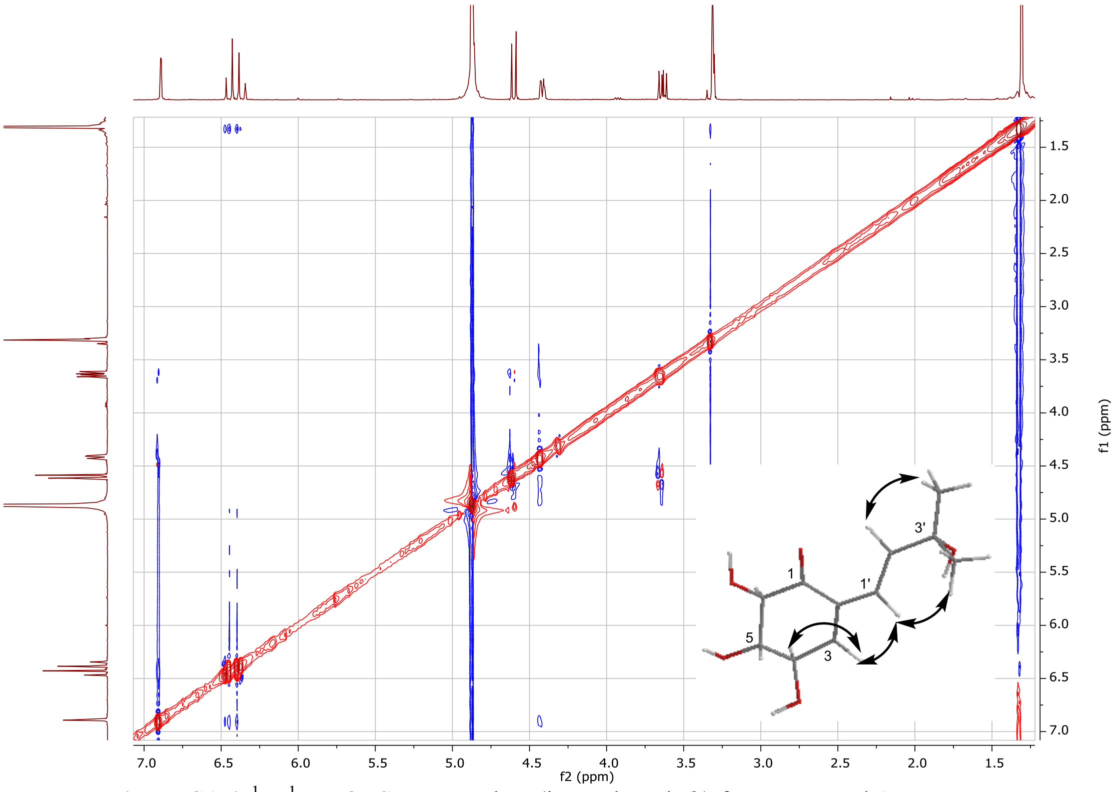

Figure S1.6. ^{1}H-^{1}H NOESY NMR data (in methanol-d_4) for compound **1**

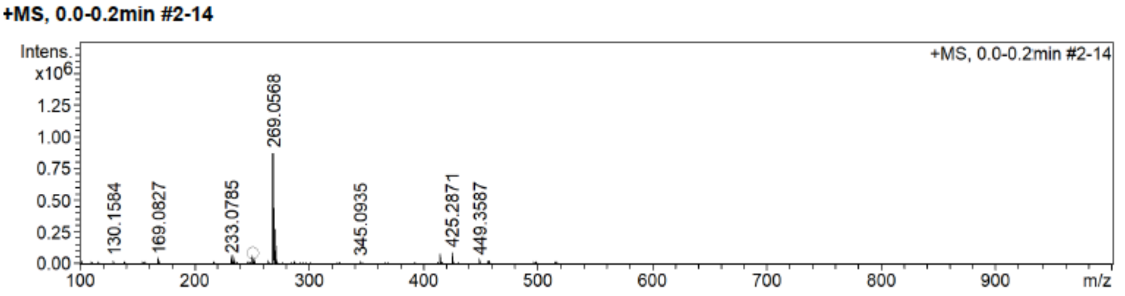

Meas. m/z	#	Ion Formula	m/z	err [ppm]	mSigma	# Sigma	Score	rdb	e⁻ Conf	N-Rule
251.0891	1	C11H16NaO5	251.0890	-0.6	401.3	1	100.00	3.5	even	ok

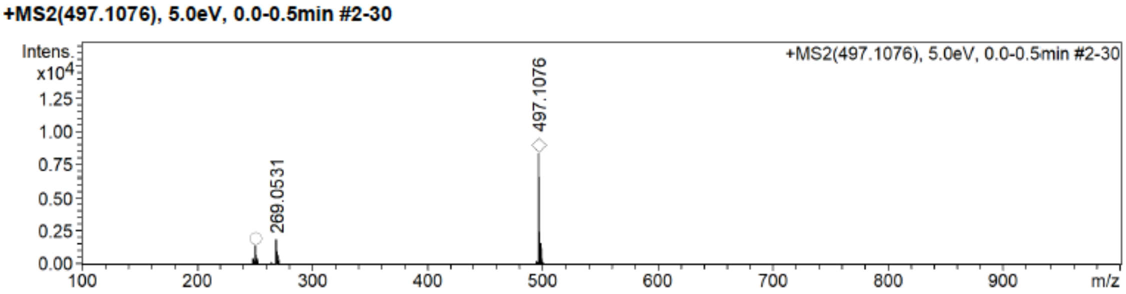

Meas. m/z	#	Ion Formula	m/z	err [ppm]	mSigma	# Sigma	Score	rdb	e⁻ Conf	N-Rule
251.0879	1	C11H16NaO5	251.0890	-4.5	149.9	1	100.00	3.5	even	ok

Figure S1.7. ESIMS (positive mode) data for compound **1**

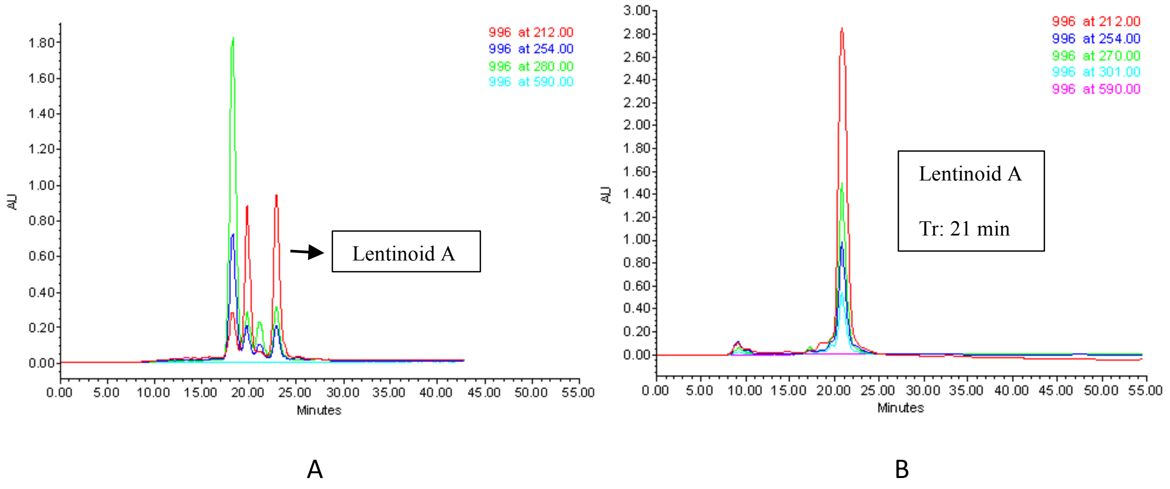

Figure S1.8. Chromatogram of compound **1**: **A)** Separation; **B)** Purity

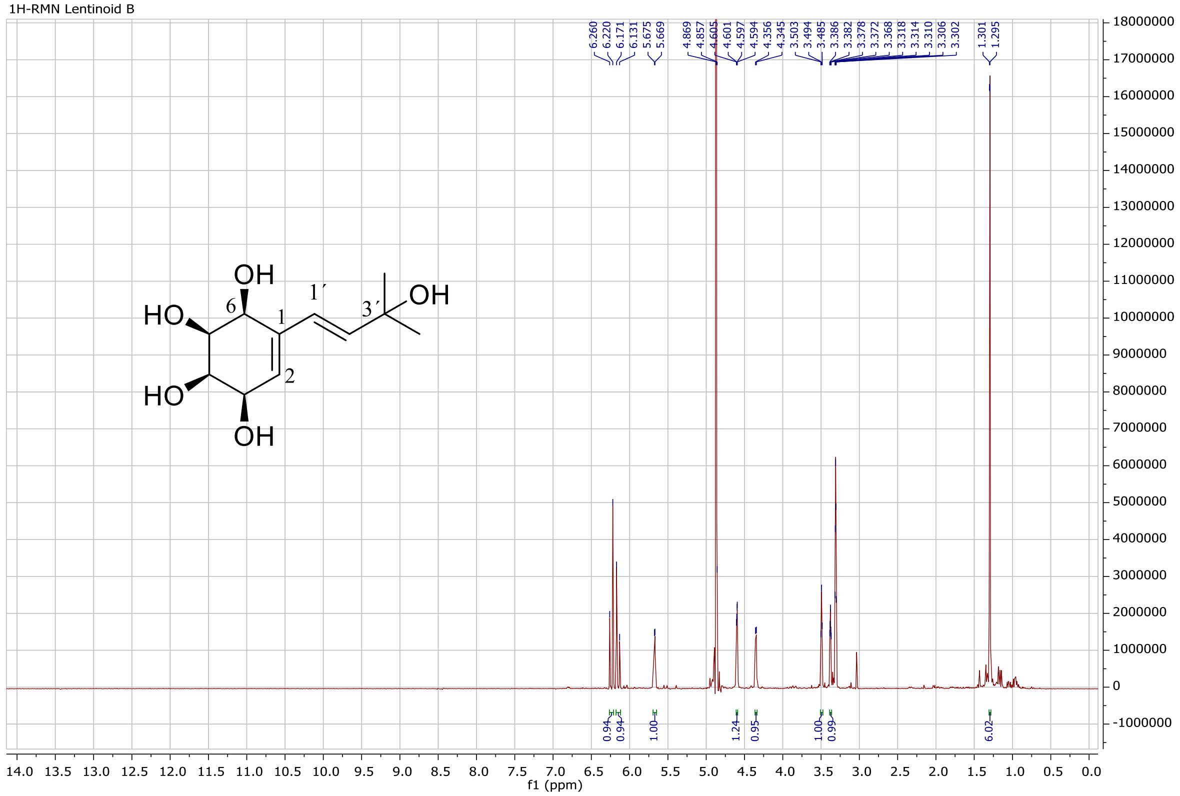

Figure S2.1. ^{1}H NMR spectra (400 MHz, methanol-d_4) of compound **2**

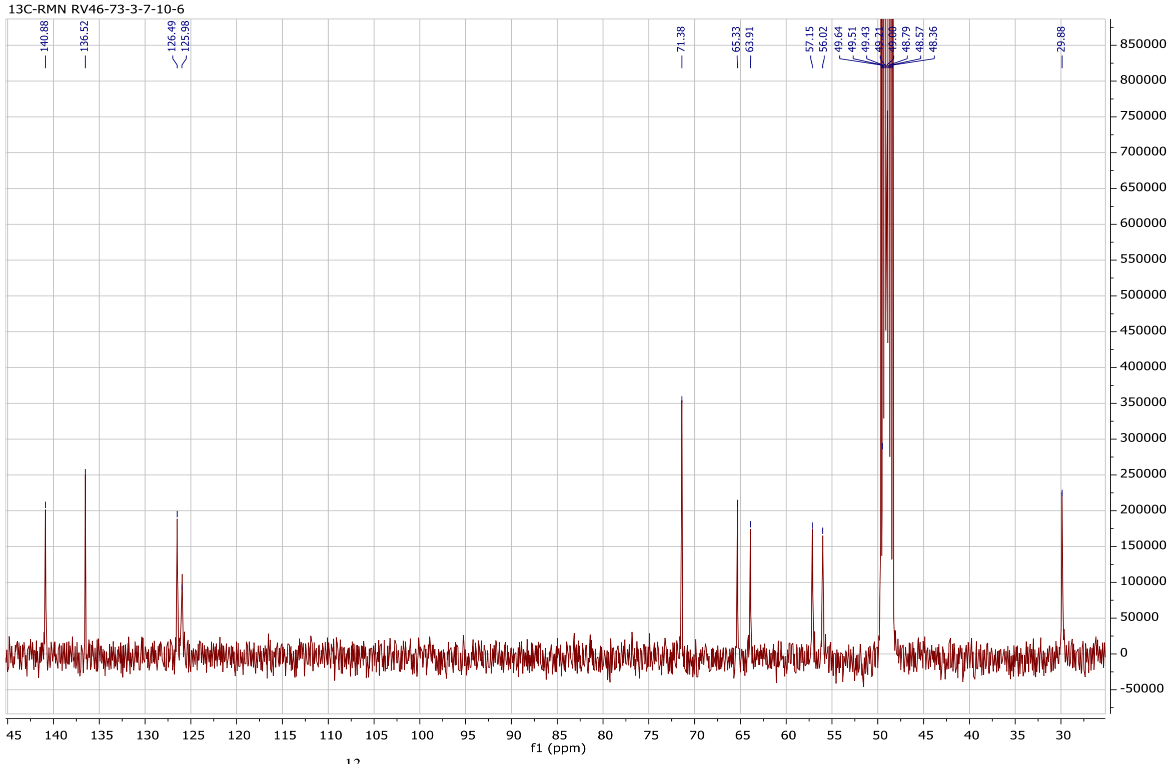

Figure S2.2. ^{13}C NMR spectra (100 MHz, methanol-d_4) of compound **2**

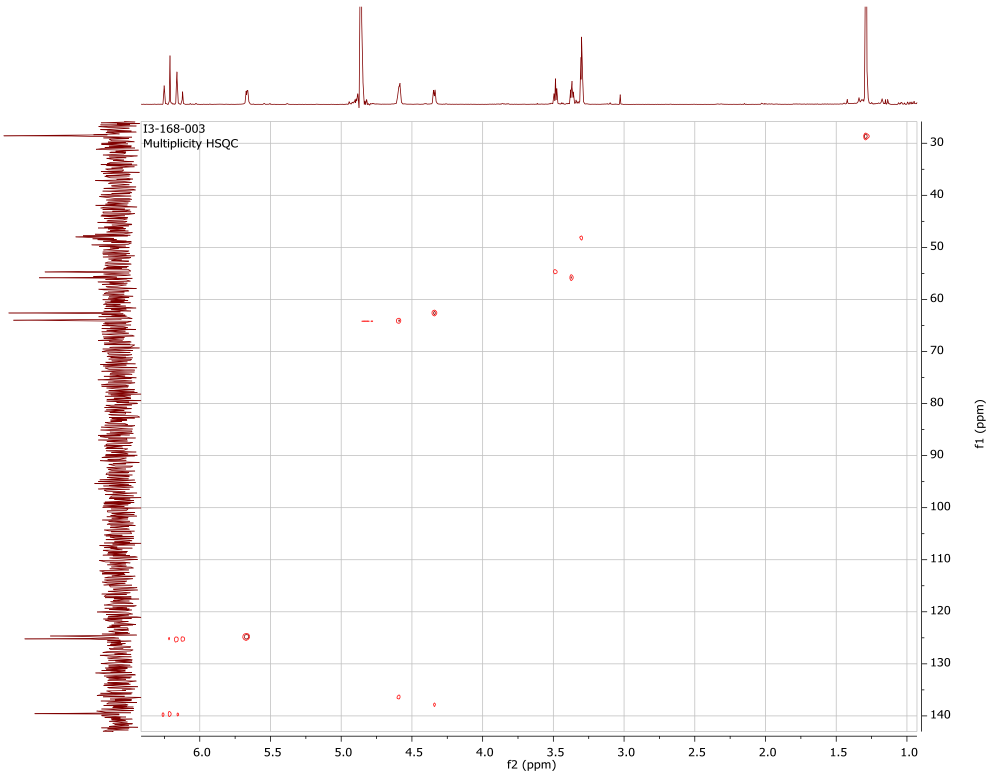

Figure S2.3. ^{1}H- ^{13}C HSQC NMR data (in methanol-d_4) of compound **2**

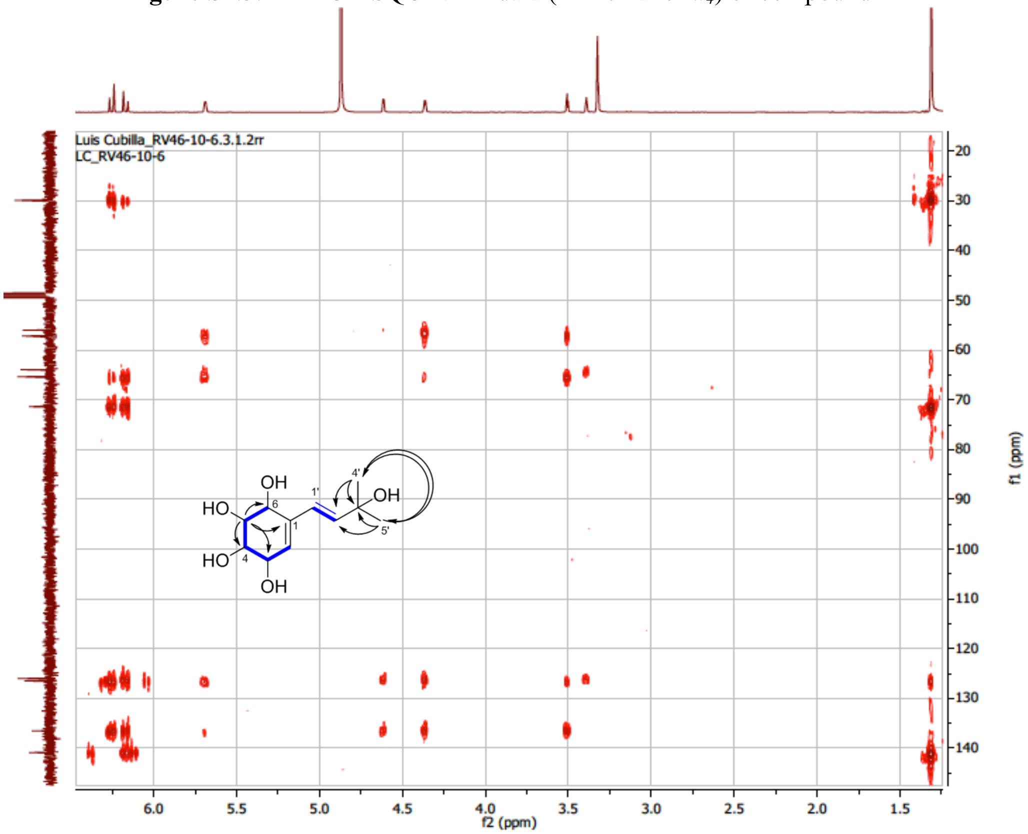

Figure S2.4. ^{1}H- ^{13}C HMBC NMR data (in methanol-d_4) of compound **2**

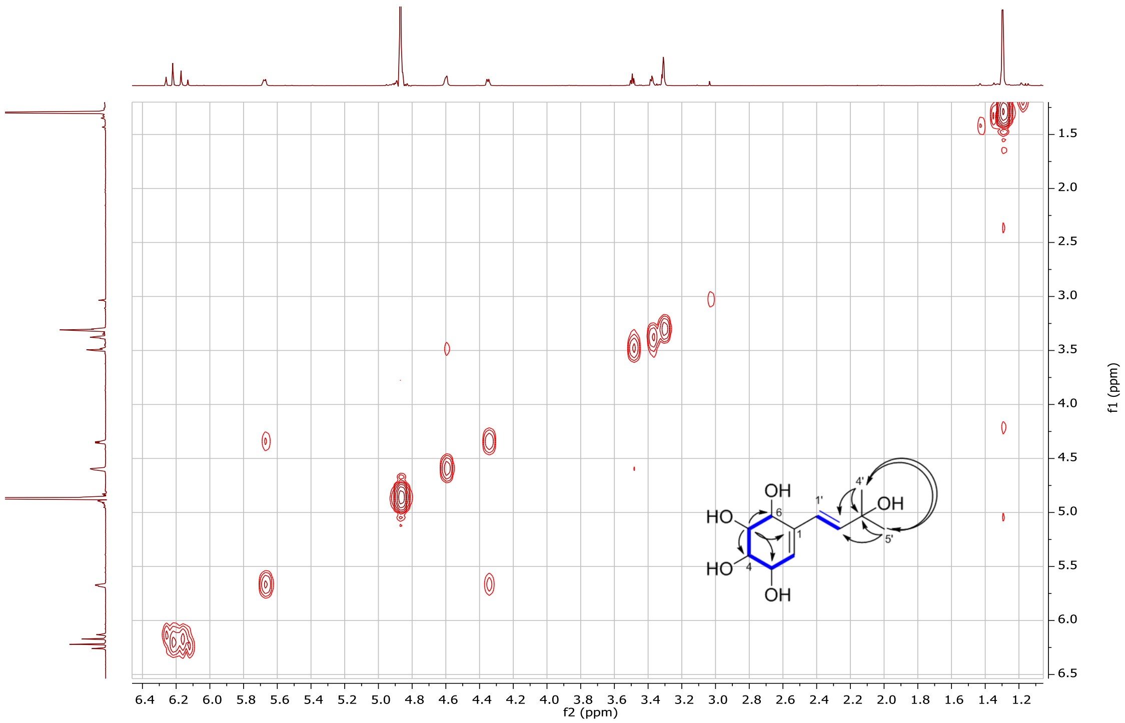

Figure S2.5. ^{1}H-^{1}H COSY NMR data (in methanol-d_4) of compound **2**

NOESY - RV46-73-3-7-10-6

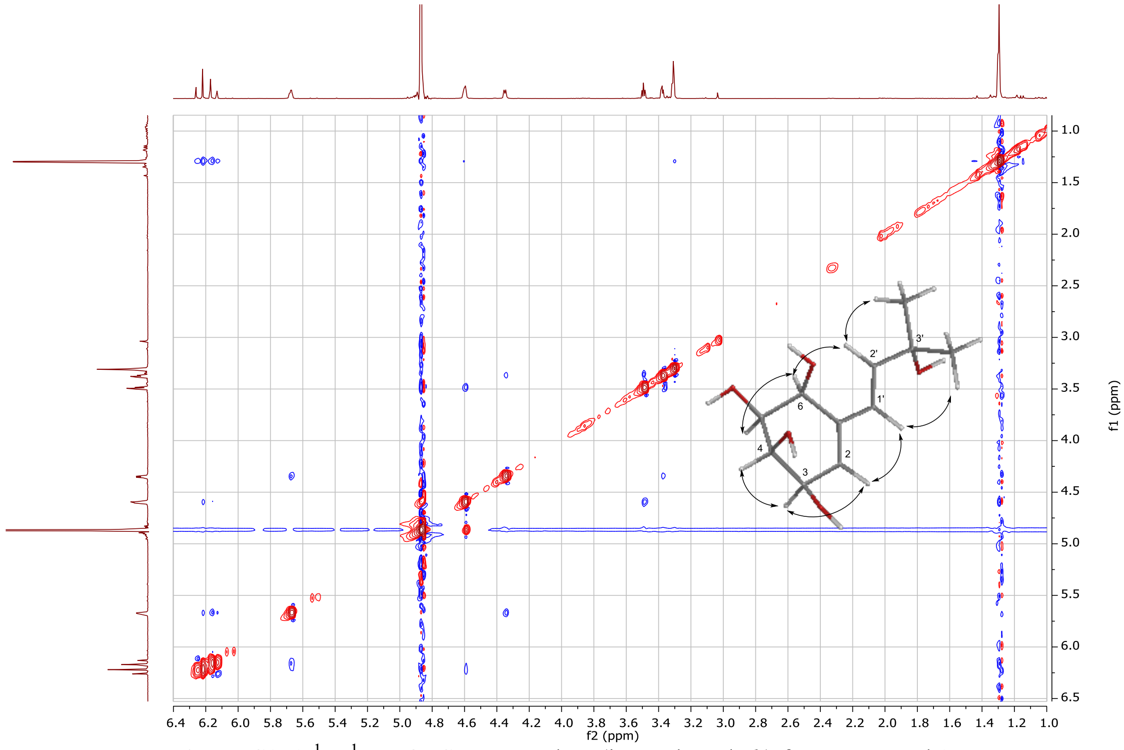

Figure S2.6. ^{1}H-^{1}H NOESY NMR data (in methanol-d_4) for compound **2**

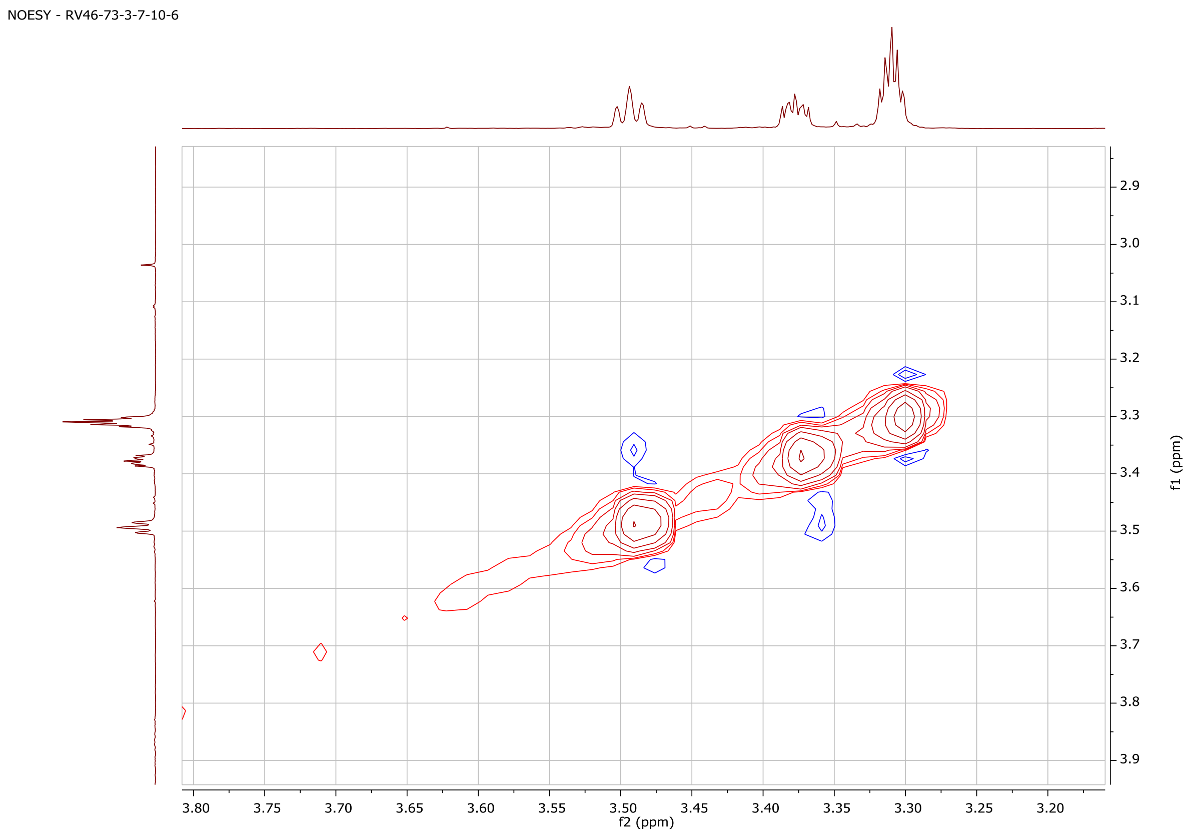

Figure S2.7. Expansion of ^{1}H-^{1}H NOESY NMR for the correlations between H4-H5 in compound **2**

+MS, 0.0-0.3min #2-15

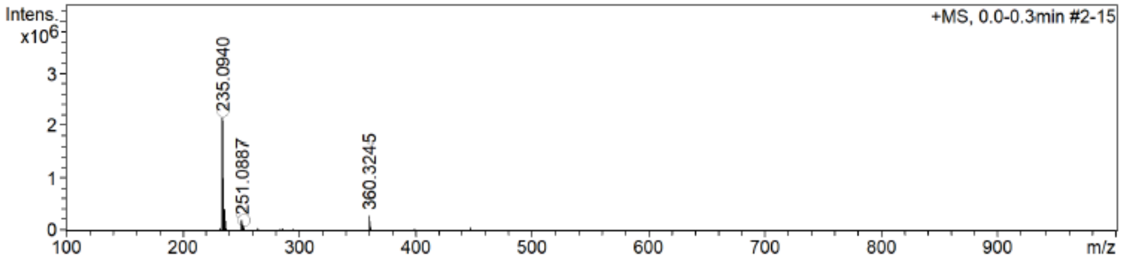

Meas. m/z	#	Ion Formula	m/z	err [ppm]	mSigma	# Sigma	Score	rdb	e⁻ Conf	N-Rule
235.0940	1	C11H16NaO4	235.0941	0.4	42.5	1	100.00	3.5	even	ok
251.0887	1	C11H16NaO5	251.0890	1.3	155.6	1	100.00	3.5	even	ok
253.1034	1	C11H18NaO5	253.1046	-4.9	19.2	1	100.00	2.5	even	ok

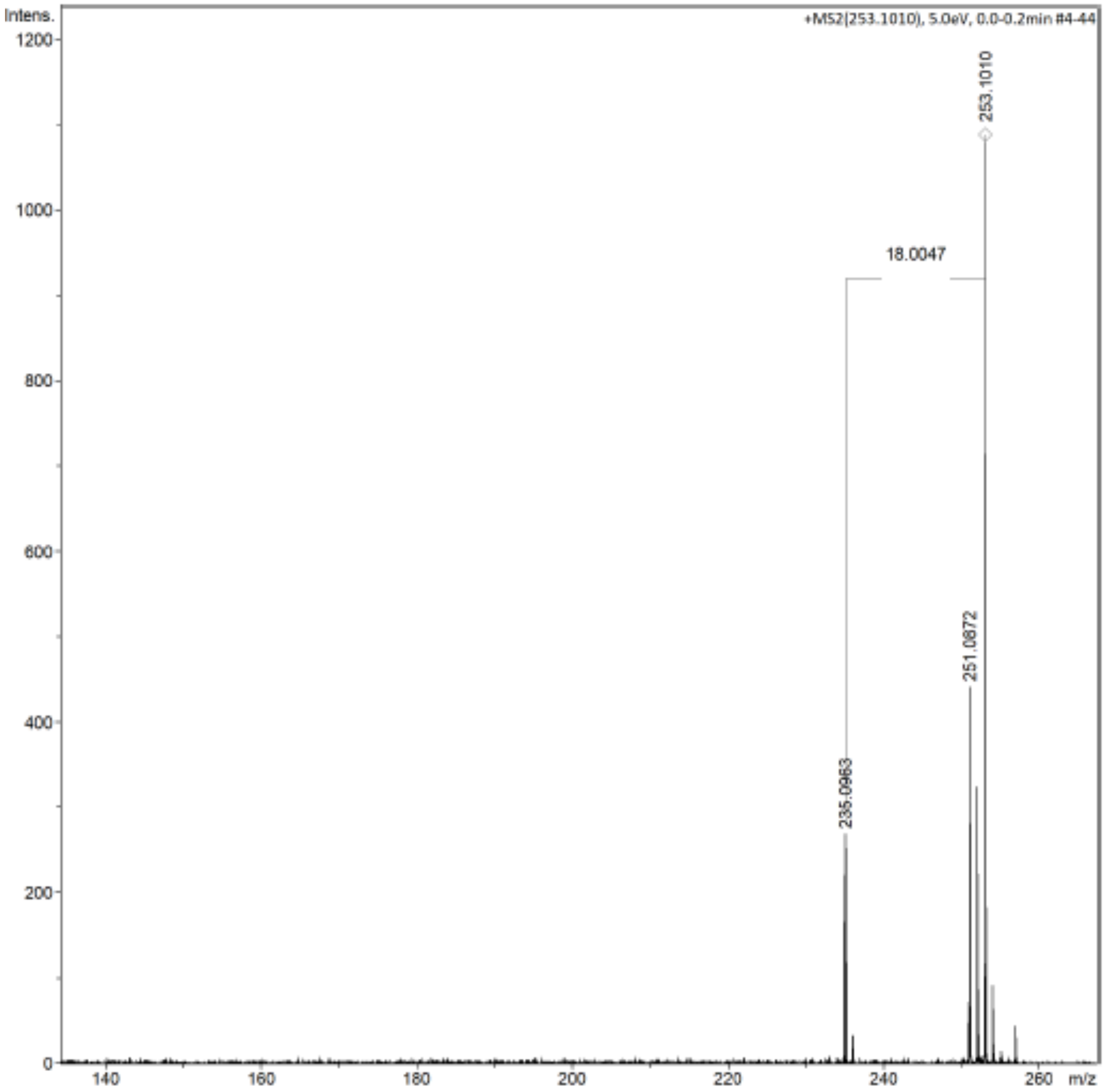

Figure S2.8. ESIMS (positive mode) data for compound **2**

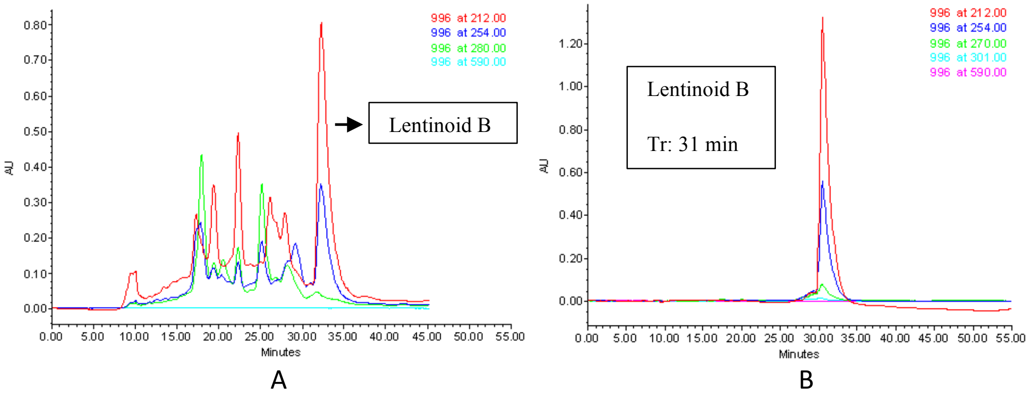

Figure S2.9. Chromatogram of compound **2**: **A)** Separation; **B)** Purity

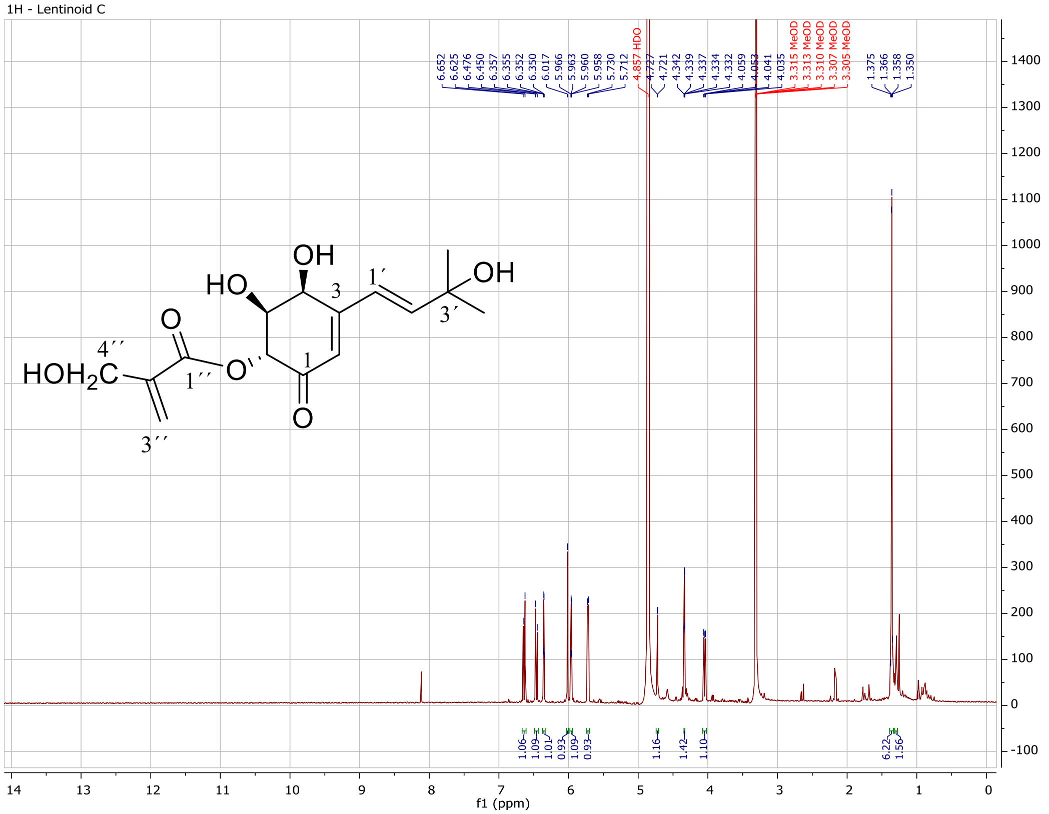

Figure S3.1. ^{1}H NMR spectra (600 MHz, methanol-d_4) of compound **3**

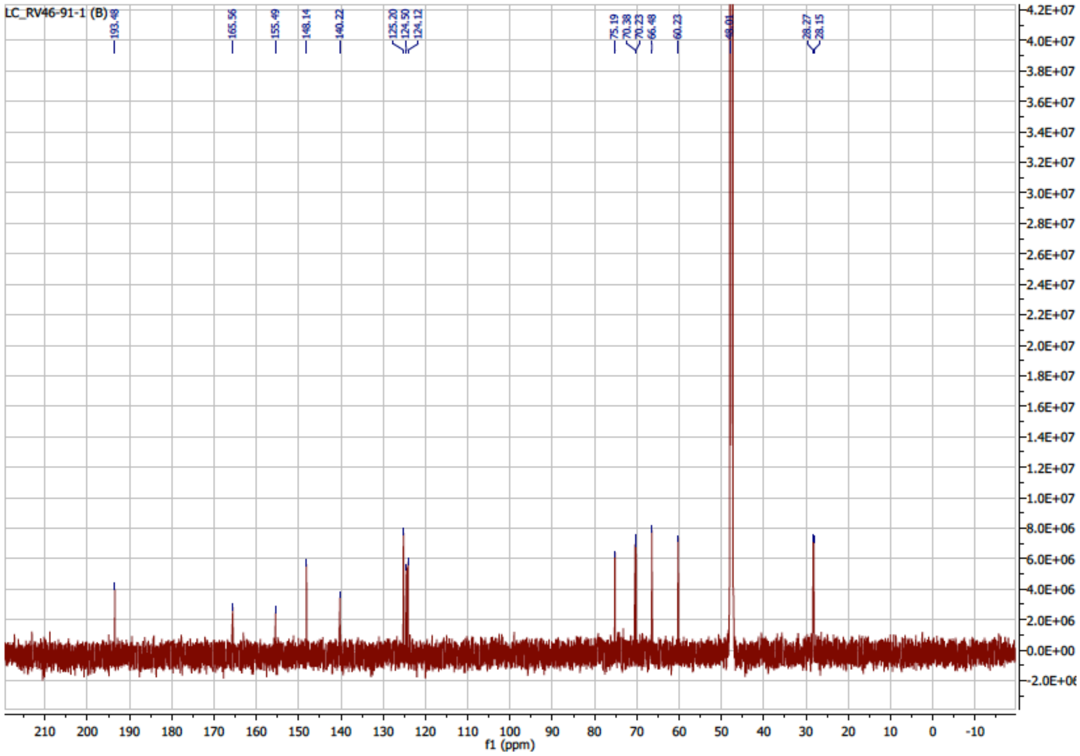

Figure S3.2. ^{13}C NMR spectra (150 MHz, methanol-d_4) of compound **3**

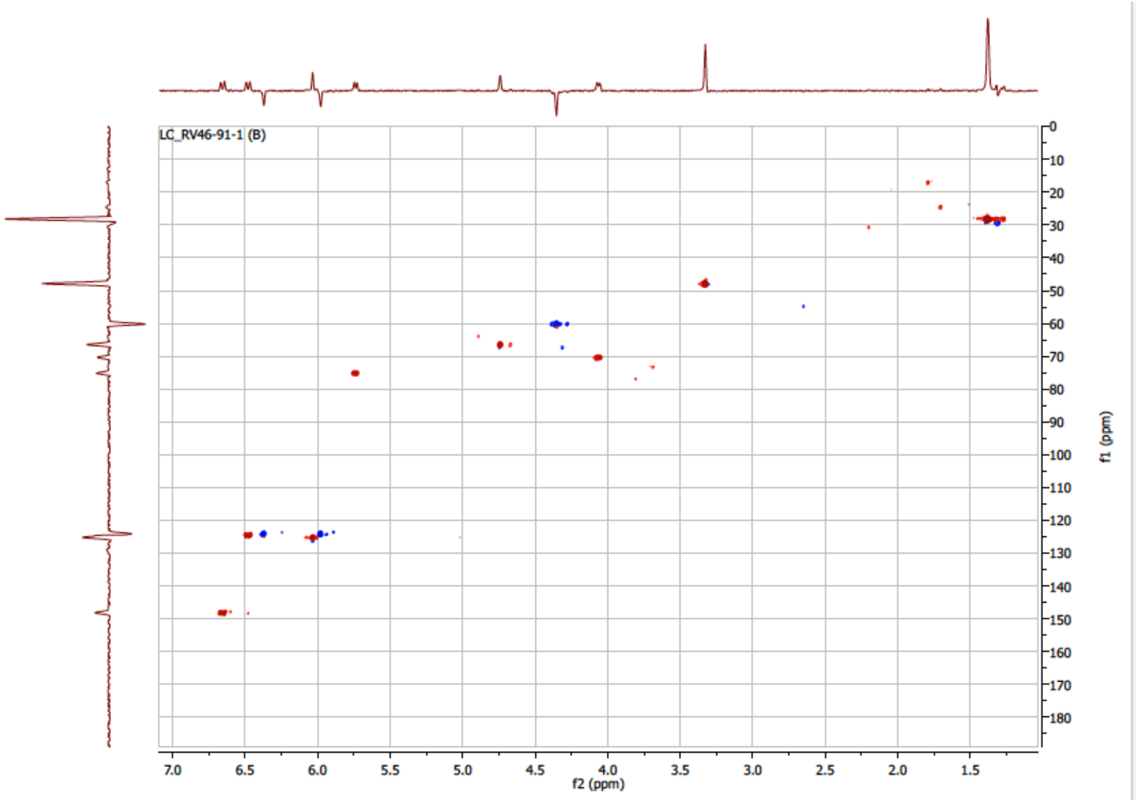

Figure S3.3. ^{1}H- ^{13}C HSQC NMR data (in methanol-d_4) of compound **3**

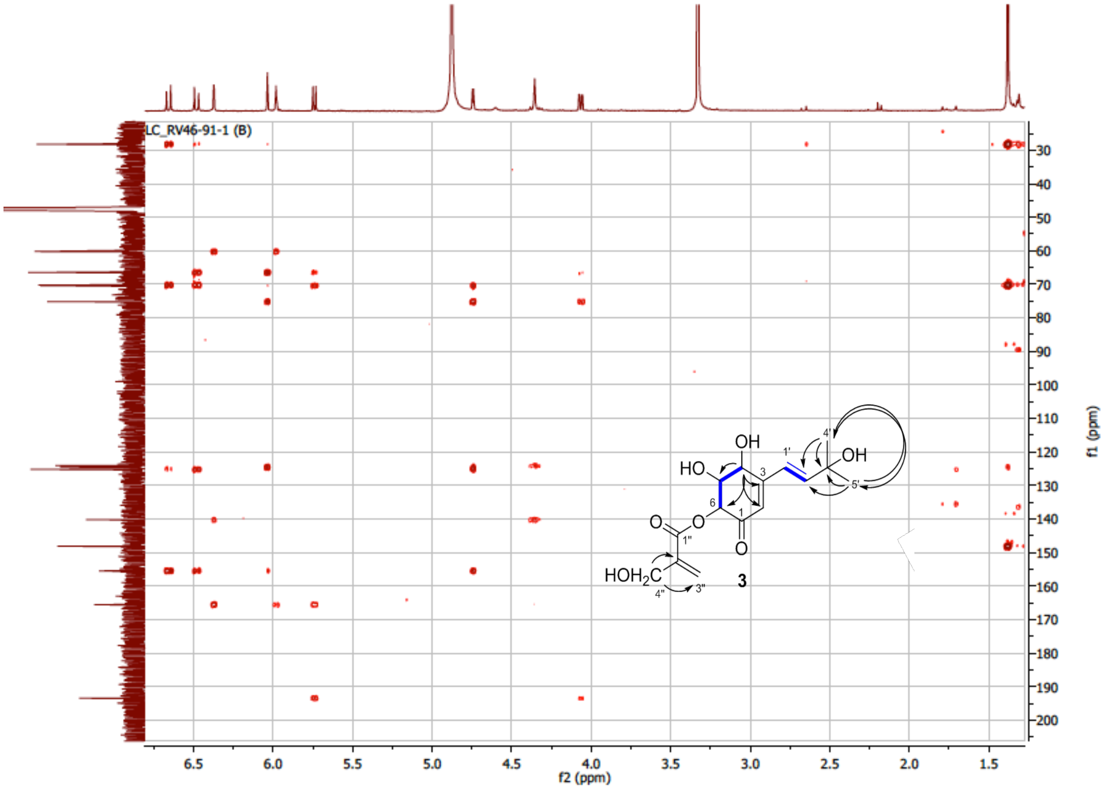

Figure S3.4. ^{1}H- ^{13}C HMBC NMR data (in methanol-d_4) of compound **3**

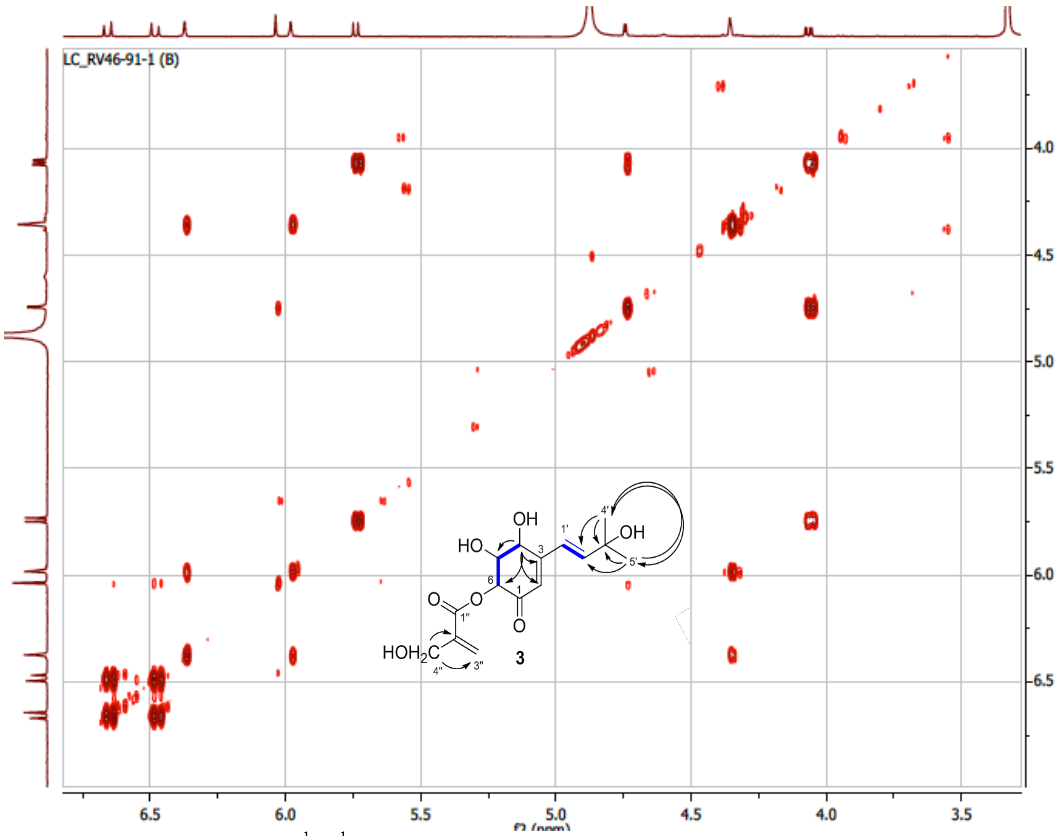

Figure S3.5. ^{1}H-^{1}H COSY NMR data (in methanol-d_4) of compound **3**

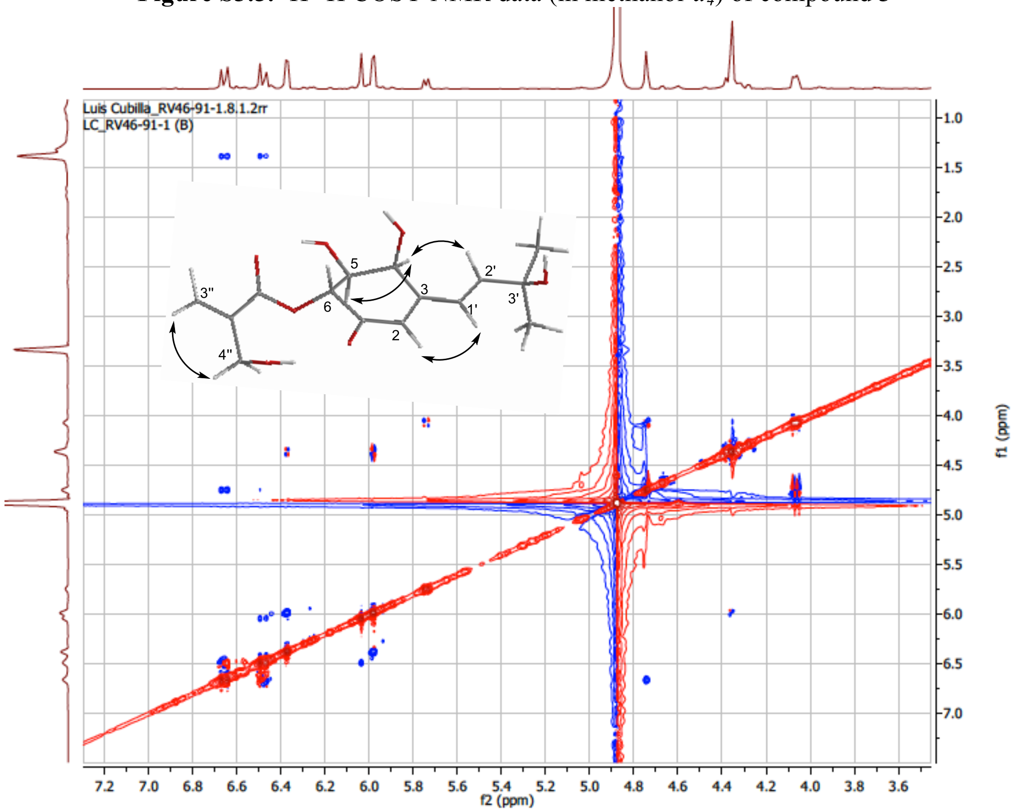

Figure S3.6. ^{1}H-^{1}H ROESY NMR data (in methanol-d_4) for compound **3**

+MS, 0.0-0.5min #2-29

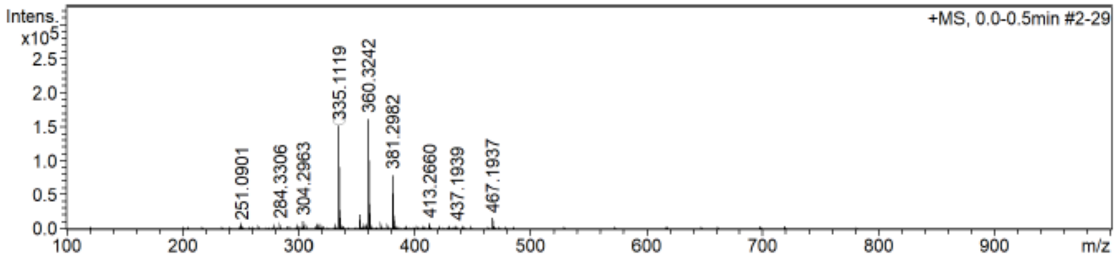

Meas. m/z	#	Ion Formula	m/z	err [ppm]	mSigma	# Sigma	Score	rdb	e⁻ Conf	N-Rule
335.1119	1	C15H20NaO7	335.1101	-5.3	8.4	1	100.00	5.5	even	ok

+MS2(335.1109), 5.0eV, 0.0-0.5min #1-29

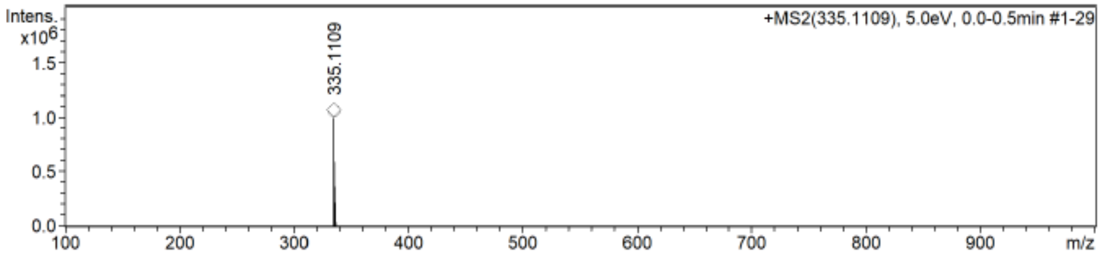

Meas. m/z	#	Ion Formula	m/z	err [ppm]	mSigma	# Sigma	Score	rdb	e⁻ Conf	N-Rule
335.1109	1	C15H20NaO7	335.1101	-2.4	119.6	1	100.00	5.5	even	ok

+MS2(647.2311), 5.0eV, 0.0-0.5min #2-29

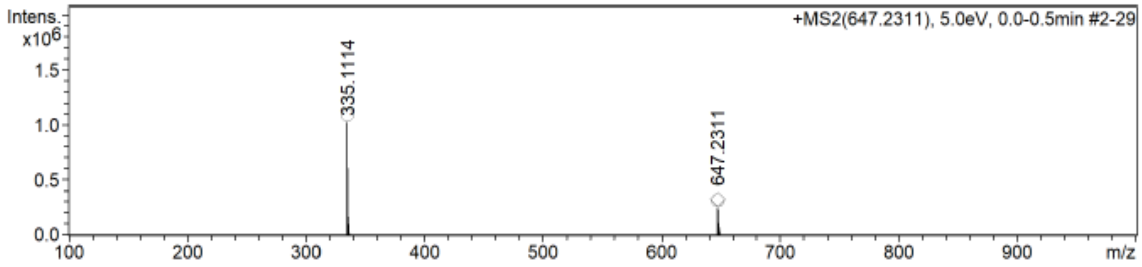

Meas. m/z	#	Ion Formula	m/z	err [ppm]	mSigma	# Sigma	Score	rdb	e⁻ Conf	N-Rule
335.1114	1	C15H20NaO7	335.1101	-3.7	9.5	1	100.00	5.5	even	ok
647.2311	1	C30H40NaO14	647.2310	-0.1	33.7	1	100.00	10.5	even	ok

Figure S3.7. ESIMS (positive mode) data for compound **3**

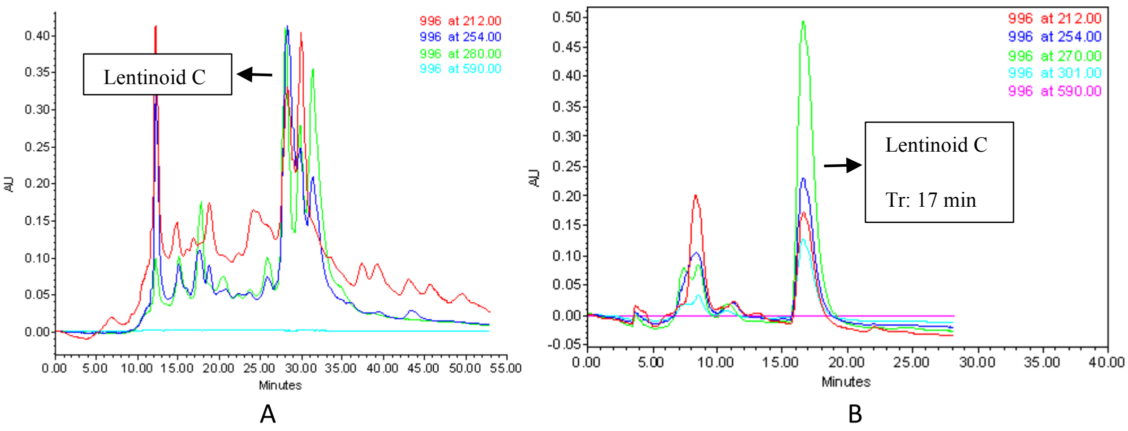

Figure S3.8. A) Normal phase HPLC separation chromatogram with semi-preparative column for compound **3. B)** Normal phase HPLC separation chromatogram with chiral pack column for compound **3**

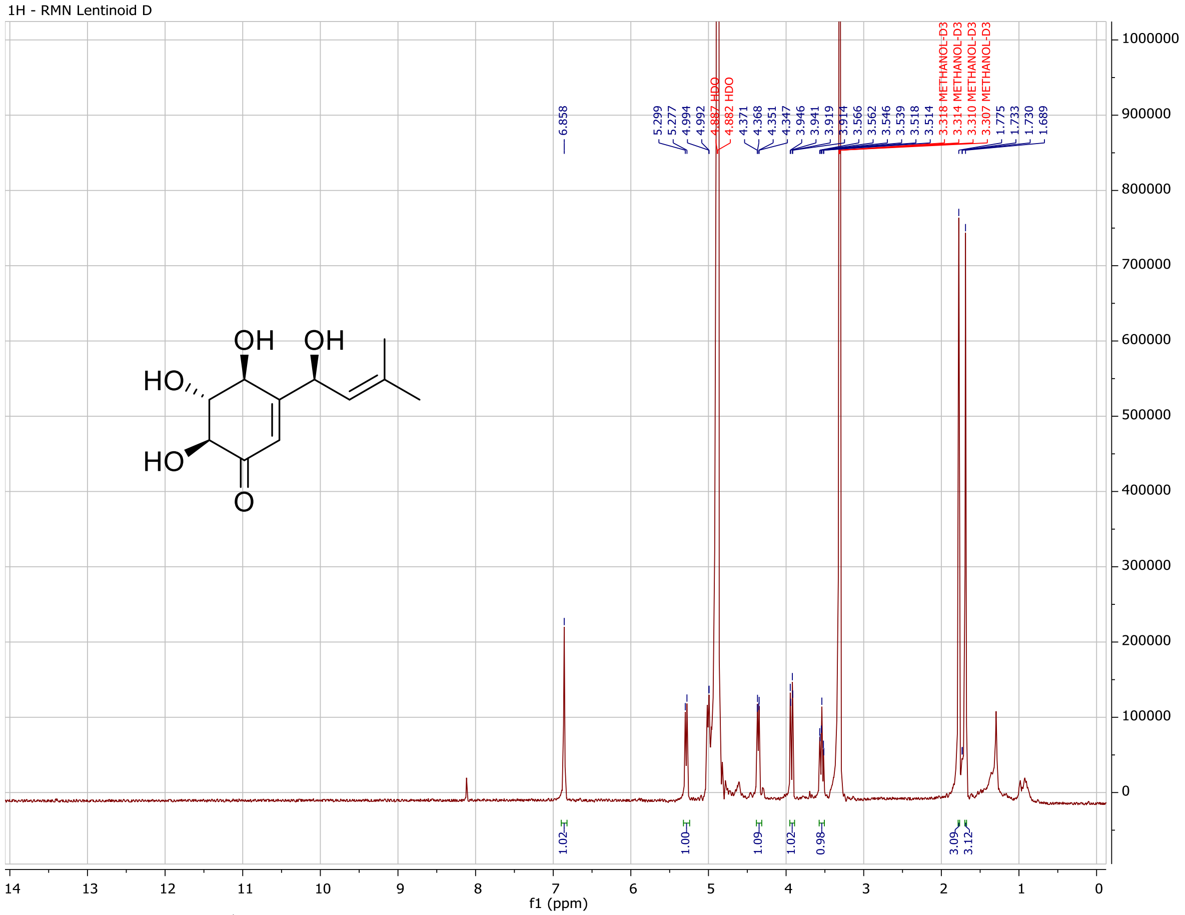

Figure S4.1. ^{1}H NMR spectra (400 MHz, methanol-d_4) of compound **4**

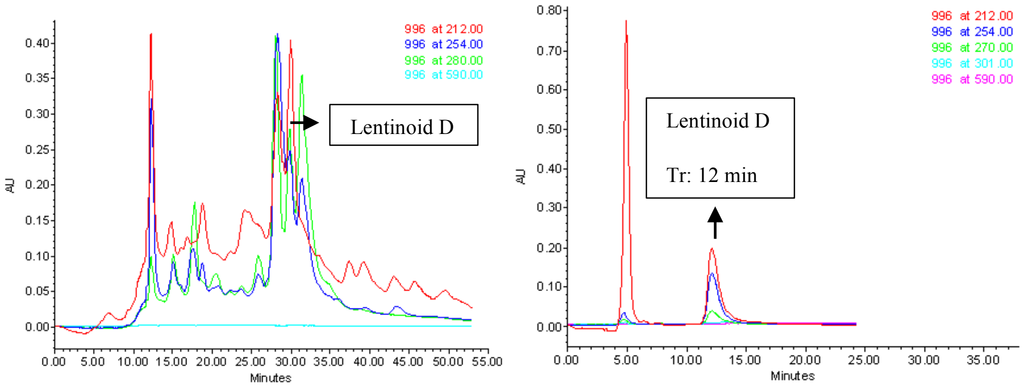

A **B**

Figure S4.2. A) Normal phase HPLC separation chromatogram with semi-preparative column for compound **4. B)** Normal phase HPLC separation chromatogram with chiral pack column for compound **4**
